# Incubation of Immune Cell Grafts With MAX.16H5 IgG1 Anti-Human CD4 Antibody Prolonged Survival After Hematopoietic Stem Cell Transplantation in a Mouse Model for Fms Like Tyrosine Kinase 3 Positive Acute Myeloid Leukemia

**DOI:** 10.3389/fimmu.2018.02408

**Published:** 2018-10-22

**Authors:** Nadja Hilger, Claudia Mueller, Lilly Stahl, Anne M. Mueller, Bianca Zoennchen, Sarah Dluczek, Christoph Halbich, Claudia Wickenhauser, Dennis Gerloff, Alexander A. Wurm, Gerhard Behre, Anna Kretschmer, Stephan Fricke

**Affiliations:** ^1^Immune Tolerance, Immunology, Fraunhofer Institute for Cell Therapy and Immunology, Leipzig, Germany; ^2^Institute for Clinical Immunology, University of Leipzig, Leipzig, Germany; ^3^Institute for Pathology, Halle University Hospital, Halle, Germany; ^4^Department of Dermatology and Venereology, University Hospital Halle, Halle, Germany; ^5^Division of Hematology and Medical Oncology, Leipzig University Hospital, Leipzig, Germany

**Keywords:** anti-human CD4 antibody MAX.16H5, hematopoietic stem cell transplantation, graft-vs.-host disease, acute myeloid leukemia, 32D-FLT3^ITD^, graft-vs.-leukemia, C3H/HeN

## Abstract

Despite the constant development of innovative therapeutic options for hematological malignancies, the gold-standard therapy regimen for curative treatment often includes allogeneic hematopoietic stem cell transplantation (HSCT). The graft-vs.-leukemia effect (GVL) is one of the main therapeutic goals that arises from HSCT. On the other hand, graft-vs.-host disease (GVHD) is still one of the main and most serious complications following allogeneic HSCT. In acute myeloid leukemia (AML), HSCT together with high-dose chemotherapy is used as a treatment option. An aggressive progression of the disease, a decreased response to treatment, and a poor prognosis are connected to internal tandem duplication (ITD) mutations in the Fms like tyrosine kinase 3 (FLT3) gene, which affects around 30% of AML patients. In this study, C3H/HeN mice received an allogeneic graft together with 32D-FLT3^ITD^ AML cells to induce acute GVHD and GVL. It was examined if pre-incubation of the graft with the anti-human cluster of differentiation (CD) 4 antibody MAX.16H5 IgG_1_ prevented the development of GVHD and whether the graft function was impaired. Animals receiving grafts pre-incubated with the antibody together with FLT3^ITD^ AML cells survived significantly longer than mice receiving untreated grafts. The observed prolonged survival due to MAX.16H5 incubation of immune cell grafts prior to transplantation may allow an extended application of additional targeted strategies in the treatment of AML.

## Introduction

Allogeneic hematopoietic stem cell transplantation (HSCT) remains a standard therapy regimen for the curative treatment of hematological malignancies (1). The graft-vs.-leukemia effect (GVL) is the main therapeutic goal that arises from HSCT [reviewed in (2)]. The leading cause of morbidity and mortality following allogeneic HSCT is graft-vs.-host disease (GVHD) (3–5), which is characterized by an imbalance of effector and regulatory mechanisms of the generated immune response (6). Alloreactive αβ T cells in the graft are regarded as the major cause for the development of GVHD in certain transplantation settings (6). Additionally, natural killer (NK) cells and/or a failure of regulatory immune pathways are potentially involved in GVHD [reviewed in (7, 8)]. Although T cell depletion prevents GVHD development, the increased risk for infections and host-vs.-graft reaction, defective cytokine production, and reduced engraftment lead to an attenuated graft-vs.-leukemia effect (GVL) [(6), reviewed in (9)]. The GVL effect can be observed together with GVHD or can occur independently (10), also due to minor histocompatibility antigens [summarized in (11)].

It is known that T and NK cells are able to recognize and destroy tumor cells by binding to various immunologically important surface markers (12). Cytotoxic T lymphocytes [CTLs, cluster of differentiation (CD) 8^+^] play an important role in anti-tumor activity by the detection of endogenous tumor antigens presented by major histocompatibility complexes (MHC) class I leading to an activation of CTLs into effector cells which promote tumor lysis [reviewed in (13)]. Anti-tumor activity of T helper cells (CD4^+^) is often impaired since the expression of MHC class II is restricted to professional antigen presenting cells, e.g., dendritic cells, macrophages, and B cells [reviewed in (14)]. Despite an expression of MHC class II on hematological cancer cells [e.g., B cell malignancies (15)], the majority of solid cancers lack MHC class II expression completely or can escape the recognition by the immune system using immune editing mechanisms (16–18). Although CD4^+^ T cell induced expression of cytokines was shown to support CTL activation [summarized in (19, 20)], immune escape and tumor progression is promoted by CD4^+^ T cell anergy (21, 22). Besides MHC class I and MHC class II (23), surface antigens like CD40, CD80, and CD86 are widely expressed on both healthy (antigen presenting cells) and malignant cells where their co-stimulatory function triggers T cells and T cell driven immune responses [reviewed in (24, 25)].

In previous work, our data showed that the short-term *ex vivo* incubation of an allogeneic graft with the non-depleting anti-human CD4 antibody MAX.16H5 IgG_1_ (murine) led to a significant GVHD reduction without negatively influencing the induced GVL effect (26). Additionally, NOD.Cg-Prkdc^scid^ IL-2rg^tm1Wjl^/SzJ (NSG) recipient mice showed a significantly increased survival after xenogeneic transplantation of human peripheral blood mononuclear cells when the graft was pre-treated with the anti-human CD4 antibody MAX.16H5 IgG_1_ *ex vivo* (27). Possible side effects emerging from the antibody treatment did not occur, most likely because a systemic administration of MAX.16H5 IgG_1_ was not required to achieve treatment success. The observation that a single administration of an anti-human CD4 antibody can downregulate GVHD development is challenging the accepted theory and practice of long-term continuous T cell suppression by systemic immunosuppressant drugs. The described anti-human CD4 antibody recognizes the first domain (D1) of the CD4 molecule, which is an Ig-like V-type domain and contains three CDR-like regions (CDR1, CDR2, CDR3) (28). In previous studies, we provided evidence that the GVHD development was significantly downregulated by using the MAX.16H5 IgG_1_ antibody (27, 29). The anti-tumor effect of MAX.16H5 IgG_1_ incubated grafts was shown to be concurrently unaffected in a murine mastocytoma model (BALB/c) (26).

Regarding these promising results, we decided to investigate whether the antibody-induced GVHD prevention and retained anti-tumor effect can be translated into an Fms like tyrosine kinase 3 (FLT3, CD135) internal tandem duplication (ITD) positive acute myeloid leukemia (AML) C3H mouse model since acute GVHD affects 45–53% of AML patients carrying FLT3 mutations (30, 31). FLT3 is involved in proliferation, survival, and differentiation processes of hematopoietic cells and in the development of B and T cells [reviewed in 32)]. The most frequent mutation detected in AML patients (approximately 30%) is the ITD mutation, which affects the juxtamembrane domain of the FLT3 receptor (class I mutation) [reviewed in 32, 33)]. Several studies connected the FLT3^ITD^ mutation to a decreased response to treatment and a poor prognosis (34–37). The significance of the FLT3 receptor and its downstream signaling pathways in AML led to the development of several inhibitory drugs (e.g., Sorafenib®, Quizartinib®, Midostaurin®) that are currently under investigation in different clinical trials [(38), reviewed in (39, 40)] or that are already EMA and FDA approved for the treatment of FLT3-positive AML (41, 42).

In this study, we investigated whether the transplantation of anti-CD4 antibody (MAX.16H5 IgG_1_) pre-incubated grafts (of CD4/DR3 transgenic donor mice) leads to an attenuated GVHD in a full murine MHC mismatch FLT3^ITD^ positive AML model. We further analyzed if the MAX.16H5 IgG_1_ antibody incubation negatively influences the graft function.

## Materials and methods

### Animals

This study was carried out in accordance with the recommendations of the guideline of the University of Leipzig animal care committee. The protocol was approved by the regional board of animal care for the district of Leipzig (State Directorate Saxony, Leipzig). For transplantation experiments, C3H/HeN and CD4/DR3 [murine (mu) CD4 knockout, human (hu) CD4, human leukocyte antigen isotype DR3 (HLA-DR3); C57Bl/6 background (43)] mice were used. C3H/HeN (male) recipient mice were purchased from Charles River, Sulzfeld Germany. CD4/DR3 donor mice were bred at the Max-Bürger-Forschungszentrum, University of Leipzig under standardized conditions. After irradiation, C3H/HeN mice were treated with antibiotics for 14 days (Baytril® 2.5% *ad us. vet*., Bayer Animal Health GmbH, Leverkusen, Germany). Survival, general condition [e.g., structure of fur, behavior (44)], and weight of the recipient animals were determined.

### Preparation of the immune cell graft and transplantation procedure

#### Bone marrow cells and splenocytes

Bone marrow cells (BMCs) and splenocytes (SpC) were dissected from CD4/DR3 mice as previously described (26). BMCs and splenocytes were either incubated with 800 μg MAX.16H5 IgG_1_ (1mg/mL, Biotest) per 1.4 × 10^8^ cells or without antibody. Antibody incubation was performed in 30 mL serum free media (RPMI 1640, GlutaMAX™-I, Gibco™, ThermoFisher SCIENTIFIC, Darmstadt, Germany) and samples were incubated in the dark at room temperature for 1 to 2 h. After incubation, BMCs and splenocytes were washed twice with phosphate buffered saline (1 × PBS, Gibco™, ThermoFisher SCIENTIFIC, Darmstadt, Germany), centrifuged at 300 × g for 10 min and finally resuspended in 1 × PBS.

#### Tumor cells

The myeloblast-like murine cell line 32D Clone 3 (ATCC® CRL-11346™) expressing either human FLT3^wt^ (wildtype) or human FLT3^ITD^ as described elsewhere (45) was kindly provided by the group of Prof. G. Behre (University of Leipzig). 32D-FLT3^ITD^ cells show human FLT3 expression with the ITD mutation. The cells were cultured as previously described (46).

#### Transplantation procedure

Before transplantation, all recipient C3H/HeN animals received a total body irradiation of 8 Gy (X-Ray, D3225, Orthovoltage; Gulmay Medical, Camberley, UK) as previously described (47). After 4 h, the mice underwent allogeneic BMC and splenocyte transplantation. Mice received either 2 × 10^7^ BMCs and 2 × 10^7^ splenocytes or 1 × 10^7^ BMCs and 3 × 10^7^ splenocytes from CD4/DR3 mice with or without previous *ex vivo* incubation with anti-human CD4 antibody MAX.16H5 IgG_1_ (murine). For co-transplantation experiments, 5 × 10^3^ 32D-FLT3^wt^ or 5 × 10^3^ 32D-FLT3^ITD^ tumor cells were added to the graft immediately before transplantation. All cells were mixed in a final volume of 150 μL sterile 0.9% NaCl (B. Braun Melsungen AG, Germany) and immediately injected intravenously into the lateral tail vein by using a syringe with integrated needle (0.3 × 8 mm, Omnican® 20, U-40-Insulin, 0.5 mL/20 I.U., B. Braun Melsungen AG, Germany). The engraftment of the transplanted cells was observed up to 56 days.

### Flow cytometry and blood sample analysis

For all flow cytometry experiments, a BD FACSCanto^TM^ II device was used as previously described (29). All grafts were analyzed by flow cytometry prior to transplantation. 1 × 10^6^ BMCs, splenocytes, and 32D-FLT3^wt^ or 32D-FLT3^ITD^ cells were stained according to the manufacturer's protocols using specific antibodies as described in the following: Staining protocol for bone marrow (BM) and splenocytes: muCD3-FITC, muCD4-PE-Cy7, muCD8-PerCP, muCD19-APC-Cy7, huCD4-APC, HLA-DR3-FITC, muMHCI H-2K(b)-PE, non-vital staining: 7AAD. Staining protocol for 32D-FLT3^ITD^ AML cells: huCD135-PE, muCD11b-APC-Cy7, F4/80-PE-Cy7, Gr1-APC, non-vital staining: 7AAD. Staining protocol for 32D-FLT3^wt^AML cells: huCD135-PE, muCD11b-APC-Cy7, non-vital staining: 7AAD. All antibodies were purchased from BD Biosciences, Heidelberg, Germany. After antibody incubation, the cells were washed twice with 1 × PBS and analyzed by flow cytometry.

Immune reconstitution and tumor growth were analyzed by flow cytometry and impedance measurement. Murine blood samples were collected weekly and supplemented with 10 μL Heparin (Heparin-Natrium-25000-ratiopharm®, 25,000 I.U./5 mL). The differential blood cell count was measured using the scil Vet abc hematology analyzer (scil animal care company, Viernheim, Germany). After plasma collection, the blood pellets were diluted in 100 μL 1 × PBS and incubated with antibodies (muCD3-FITC, muCD4-PE-Cy7, muCD8-PerCP, muCD19-APC-Cy7, huCD4-APC, HLA-DR3-FITC, muMHCI H-2K(b)-PE, huCD135-PE) according to the manufacturer's protocols. After incubation, each sample was incubated with 1 mL BD FACS™ lysing solution (BD Biosciences, Heidelberg, Germany) for 10 minutes to remove erythrocytes. Subsequently, the cells were washed twice with 1 × PBS. The cell pellets were resuspended in 100 μL 1 × PBS and analyzed by flow cytometry. All samples were measured and analyzed using FACS Canto BD^TM^ II and BD FACSDIVA^TM^ Software (BD Biosciences, Heidelberg, Germany) as previously described (26).

### TUNEL assay

The detection of apoptotic cells was performed using the TdT-mediated dUTP-biotin nick end labeling (TUNEL) assay (ApopTag®, Merck KGaA, Darmstadt, Germany) for light microscopy as already described in previous studies (27) and by Loo et al. (48). Accurate cell numbers in TUNEL slides were determined by image thresholding using ImageJ software (v1.46r, NIH, USA). The channels for red, blue and green were separated for better detection of apoptotic cells. The red area in the blue channel was analyzed with a pre-determined threshold for all images. The ratio of the counts in the red area (i.e., structures connected over several pixels indicating apoptotic cell nuclei) to the total marked area (counts/cm^2^) was analyzed. Five individual visual fields of one histological slide of the gut of each mouse were randomly chosen and microscopically recorded in 10 × magnification as previously described (27).

### Data acquisition and statistical tests

BD FACSCanto™ II Flow Cytometry System with FACS Diva 6.1.3 software (BD Biosciences Heidelberg, Germany), scil Vet abc (scil animal care co. GmbH, Viernheim, Germany), ImageJ 1.46 (NIH, open source), and AU480® Chemistry Analyzer (Beckman Coulter, Krefeld, Germany) were used to acquire the data. Limit values stated in the following comply with scilVet standards. Weight and clinical score were determined by blinded employees of the Medical Experimental Center of University of Leipzig. Statistical analyzes and graphics were created using SigmaPlot 11.0/SYSTAT 3.5 software (Systat Software GmbH, Erkrath, Germany) and GraphPad Prism 5 (v5.02, GraphPad Software Inc., La Jolla, CA, USA). The results are represented as mean ± standard deviation or median and percentiles. *P*-values were determined by Wilcoxon rank sum test or Student's *t*-test depending on prior Shapiro–Wilk test for normality and Levene test for equal variances (49) with a type one error α = 0.05 using SigmaPlot/SYSTAT and R version 3.4.2 (50) if not marked otherwise. Log-Rank tests and pair-wise multiple comparisons of means (Holm-Sidak method) were used for the analysis of Kaplan-Meier survival curves. A *P*-value < 0.05 indicates statistical significant differences. The term “significant” stands for “statistically significant” in all cases. Adjustment for multiple comparisons was performed using the Bonferroni method controlling the family–wise error rate if not stated otherwise. The apoptotic cell count analysis was performed using multifactorial variance analysis in R version 3.4.2 (50) with the packages multcomp (51) and userfriendlyscience for Games–Howell tests (52), and GraphPad Prism 5 (two-way ANOVA with Tukey *post-hoc* test). Partial eta-squared, calculated with the DescTools package (53), and f were used as parameters for the effect size. Nonparametric *post-hoc* testing after Kruskal-Wallis test was performed with Nemenyi tests for manual comparisons, designed using packages coin (54, 55) and multcomp (51), in the case of at least 4 measurements per group and time point or Dunn's test using the FSA package (56) otherwise. *P*-values were adjusted according to the conservative Bonferroni method adapting for the large number of comparisons. Group differences in the figures are represented by asterisks with ^*^meaning *P* < 0.05, ^**^P < 0.01, and ^***^P < 0.001.

## Results

### Prolonged survival of mice by pre-incubation of allogeneic transplants with the anti-human CD4 antibody MAX.16H5 IgG_1_

**Figure 1:**
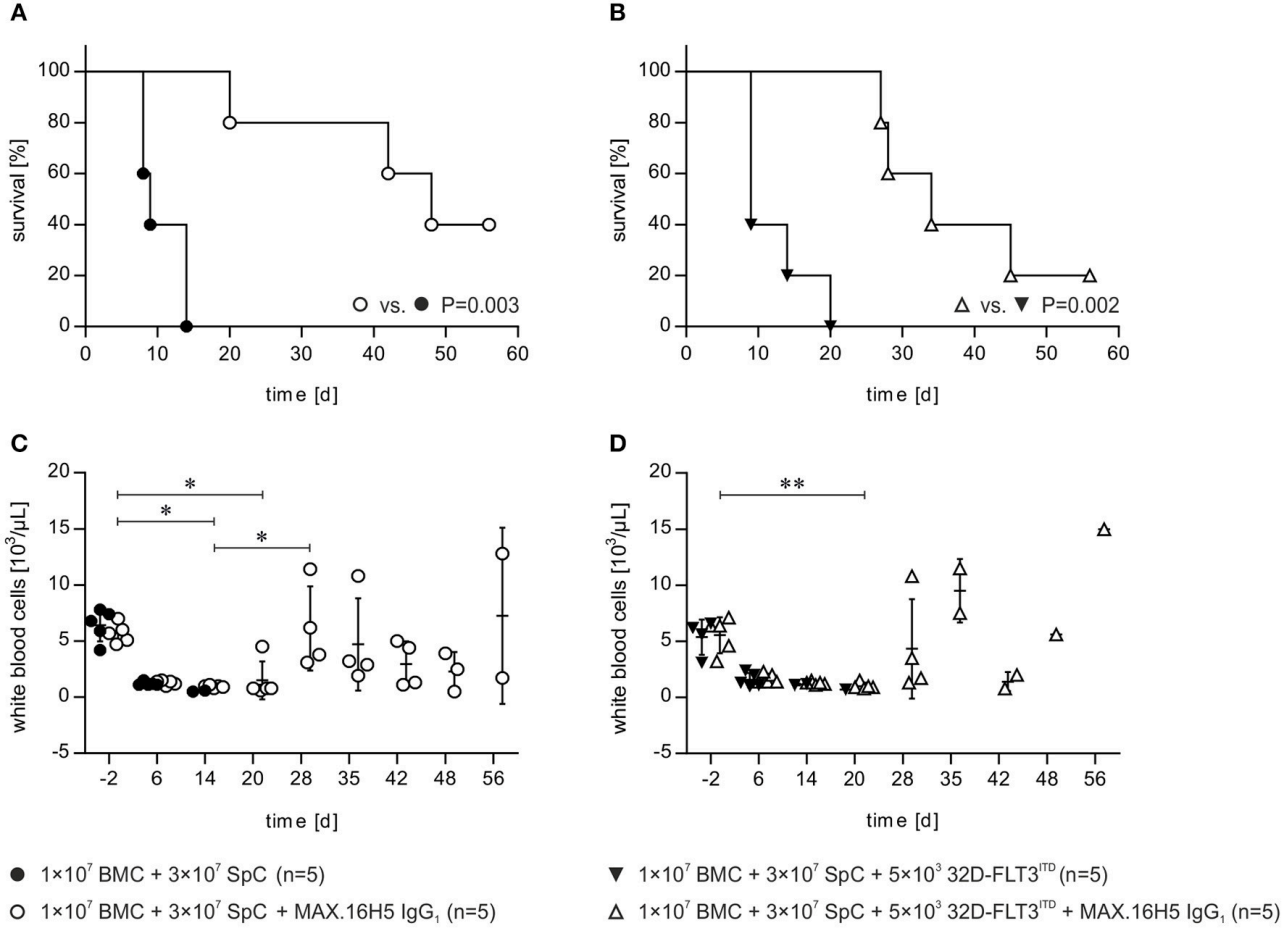
Short-term pre-incubation of bone marrow and spleen cell grafts with MAX.16H5 IgG_1_ antibody prolonged survival of C3H/HeN recipient mice after allogeneic transplantation either with or without co-transplantation of 32D-FLT3^ITD^ AML cells. All C3H/HeN mice received 1 × 10^7^ bone marrow cells (BMC) and 3 × 10^7^ spleen cells (SpC) as an allogeneic transplant in these experiments. **(A)** Survival of recipient mice after allogeneic transplantation. The grafts were pre-incubated either with MAX.16H5 IgG_1_ or without antibody before administration to the recipients. C3H/HeN animals receiving a MAX.16H5 IgG_1_ pre-incubated graft (*n* = 5, open circles) showed a significantly prolonged survival compared to animals receiving an untreated graft (*n* = 5, closed circles, *P* = 0.003; Log-Rank test). **(B)** Survival of C3H/HeN recipient mice receiving short-term pre-incubated (with or without MAX.16H5 IgG_1_ antibody) grafts together with 5 × 10^3^ 32D-FLT3^ITD^ AML cells. C3H/HeN animals receiving a MAX.16H5 IgG_1_ pre-incubated graft together with 32D-FLT3^ITD^ cells (*n* = 5, open triangle) showed a statistically significant prolonged survival compared to animals receiving an antibody untreated graft (*n* = 5, closed triangle, *P* = 0.002; Log-Rank test). **(C)** Reconstitution of white blood cells in the blood of C3H/HeN mice which received either MAX.16H5 IgG_1_ pre-incubated grafts (*n* = 5, open circle) or grafts without prior antibody incubation (*n* = 5, closed circles). **(D)** White blood cell counts in the blood of C3H/HeN mice receiving bone marrow and spleen cell grafts either pre-incubated with MAX.16H5 IgG_1_ antibody (*n* = 5, open triangle) or without antibody pre-incubation (*n* = 5, closed triangles) is depicted. The blood sample data in **(C,D)** were obtained by electrical impedance measurement using a hematology analyzer. The error bars represent time-point specific means ± standard deviations. Nemenyi *post-hoc* tests with Bonferroni correction were used for comparative statistics. *P < 0.05, **P < 0.01.

The development of GVHD and the effects of the anti-human CD4 antibody MAX.16H5 IgG_1_ were compared between recipient mice receiving 1 × 10^7^ BMCs and 3 × 10^7^ splenocytes incubated with MAX.16H5 IgG_1_ (*n* = 5) and mice receiving a control graft, which was not incubated with the antibody (*n* = 5) (Figure 1). At the end of the experiment (day 56 following transplantation), the survival rate of recipient animals receiving a MAX.16H5 IgG_1_ pre-incubated graft was 40% (*n* = 2/5) (Figure 1A). Recipient animals receiving an untreated graft died significantly earlier (*n* = 0/5, *P* = 0.003, Log-Rank test) between days 8 and 14 after transplantation (Figure 1A). The analyses of leukocytes (white blood cells, WBCs), lymphocytes, monocytes and granulocytes were performed at different time points throughout the experimental course and results are shown in Table 1 and Figure S1. Decreased leukocyte counts were observed in animals receiving untreated grafts (Figure 1C and Table 1). The WBC counts in animals receiving a MAX.16H5 IgG_1_ pre-incubated graft dropped after transplantation (day −2 vs. day 14, *P* = 0.015; day −2 vs. day 20, *P* = 0.020; Nemenyi tests) and subsequently increased until the end of the experiment (day 56; day 14 vs. day 28, *P* = 0.042, Nemenyi test). Mice receiving a graft without antibody pre-incubation showed decreased lymphocyte (Figure S1A), monocyte (Figure S1B), and granulocyte counts (Figure S1C). From day 14 on, lymphocyte, monocyte, and granulocyte cell counts were below the systems detection limit. In summary, transplanted mice receiving the MAX.16H5 IgG_1_ pre-incubated graft initially showed a decrease in lymphocyte, monocyte, and granulocyte counts followed by their recovery until the end of the experiment (Table 1, Figure S1). Blood sample analysis was also performed at the respective endpoints but no changes in the tendency of these parameters were obtained (data not shown).

**Table 1:** Blood sample analysis (WBCs, lymphocytes, monocytes, and granulocytes) of C3H/HeN mice after transplantation of 1 × 10^7^ bone marrow cells (BMC) and 3 × 10^7^ spleen cells (SpC) with or without MAX.16H5 IgG_1_ pre-incubation in combination with or without 5 × 10^3^ 32D-FLT3^ITD^ cells at different time points.

**Group**	**Blood parameters [10^3^/μL]**	**Day**								
		**−2**	**6**	**14**	**20**	**28**	**35**	**42**	**48**	**56**
1 × 10^7^ BMC + 3 × 10^7^ SpC	WBC	6.4 ± 1.4	1.2 ± 0.2	0.6 ± 0.1	–	–	–	–	–	–
	Lymphocytes	4.9 ± 1.0	0.6 ± 0.1	–	–	–	–	–	–	–
	Monocytes	0.4 ± 0.1	0.0 ± 0.0	–	–	–	–	–	–	–
	Granulocytes	1.1 ± 0.4	0.6 ± 0.1	–	–	–	–	–	–	–
1 × 10^7^ BMC + 3 × 10^7^ SpC + MAX.16H5 IgG_1_	WBC	5.7 ± 0.9	1.3 ± 0.2	1.0 ± 0.1	1.5 ± 1.7	6.1 ± 3.8	4.7 ± 4.1	3.0 ± 2.0	2.3 ± 1.7	7.3 ± 7.8
	Lymphocytes	4.6 ± 0.6	0.8 ± 0.2	0.5	3.3	3.5 ± 1.9	2.4 ± 1.7	1.6 ± 1.1	1.7 ± 0.3	3.7 ± 4.0
	Monocytes	0.3 ± 0.1	0.0 ± 0.0	0.0	0.1	0.3 ± 0.2	0.3 ± 0.2	0.2 ± 0.2	0.2 ± 0.1	0.7 ± 0.8
	Granulocytes	1.1 ± 0.2	0.6 ± 0.1	0.6	1.1	2.4 ± 1.8	2.1 ± 2.3	1.2 ± 0.8	1.4 ± 0.6	2.9 ± 3.0
1 × 10^7^ BMC + 3 × 10^7^ SpC + 5 × 10^3^ 32D-FLT3^ITD^	WBC	5.4 ± 1.6	1.6 ± 0.6	1.2 ± 0.1	0.7	–	–	–	–	–
	Lymphocytes	4.2 ± 1.3	0.9 ± 0.4	0.7 ± 0.1	–	–	–	–	–	–
	Monocytes	0.3 ± 0.1	0.0 ± 0.1	0.1 ± 0.1	–	–	–	–	–	–
	Granulocytes	0.9 ± 0.2	0.8 ± 0.2	0.5 ± 0.1	–	–	–	–	–	–
1 × 10^7^ BMC + 3 × 10^7^ SpC + MAX.16H5 IgG_1_ + 5 × 10^3^ 32D-FLT3^ITD^	WBC	5.5 ± 1.6	1.7 ± 0.4	1.3 ± 0.1	1.0 ± 0.3	4.3 ± 4.4	9.5 ± 2.8	1.4 ± 0.8	5.6	15.0
	Lymphocytes	4.2 ± 1.3	1.0 ± 0.3	0.7 ± 0.1	0.9 ± 0.3	2.4 ± 2.5	4.6 ± 1.7	1.1	3.3	8.9
	Monocytes	0.3 ± 0.1	0.0 ± 0.0	0.0 ± 0.1	0.0 ± 0.0	0.2 ± 0.2	0.6 ± 0.2	0.2	0.5	0.9
	Granulocytes	1.1 ± 0.3	0.6 ± 0.1	0.5 ± 0.0	0.4 ± 0.1	1.8 ± 1.8	4.4 ± 0.9	0.7	1.8	5.2

*Values are depicted as mean ± standard deviation. In mice receiving an untreated graft, all cell counts decreased after transplantation and did not reconstitute until the end of the experiment. The parameters in the groups receiving a MAX.16H5 IgG_1_ pre-incubated graft declined similarly after transplantation but mostly reached baseline values at the end of the experiment*.

Furthermore, it was examined whether the *ex vivo* treatment of BMCs and splenocytes with MAX.16H5 IgG_1_ prevents GVHD and prolongs the survival of recipient animals in a mouse model of FLT3^ITD^ positive AML. In initial experiments, the survival rate was 80% (*n* = 4/5) when 2 × 10^7^ BMCs and 2 × 10^7^ splenocytes were pre-incubated with MAX.16H5 IgG_1_ and co-transplanted with 5 × 10^3^ 32D-FLT3^wt^ AML cells (FLT3 wild type) (Figure S2A). All mice transplanted with an untreated graft and 5 × 10^3^ 32D-FLT3^wt^ AML cells survived (*n* = 5/5), but in two out of five animals, increased human CD135^+^ counts indicated tumor cell engraftment (Figure S2B). In contrast, no tumor engraftment was observed in mice receiving the antibody pre-incubated graft together with 32D-FLT3^wt^ AML cells over the experimental period (Figure S2B). Next, it was examined if MAX.16H5 IgG_1_ pre-incubation of an allogeneic graft improves the survival in an FLT3^ITD^ mutated AML setting. The mice were transplanted with 2 × 10^7^ BMCs and 2 × 10^7^ splenocytes incubated with or without MAX.16H5 IgG_1_ antibody and 5 × 10^3^ 32D-FLT3^ITD^ mutated AML cells. The mice receiving an antibody pre-incubated graft survived until day 35, whereas the mice without a pre-treated graft died within 18 days after transplantation (Figure S2A). On day 5 after allogeneic transplantation, the flow cytometric analysis revealed increased human CD135^+^ counts in both groups' blood samples with one mouse transplanted with an untreated graft showing the highest number. The human CD135^+^ counts in the blood samples from all animals decreased to their initial value until day 13 after transplantation (Figure S2B).

To investigate if increased human CD4^+^ T cell counts result in a prolonged survival in FLT3^ITD^ AML co-transplanted mice, splenocyte graft cell concentrations were increased from 2 × 10^7^ in the initial experiments to 3 × 10^7^. Additionally, the mice received 1 × 10^7^ BMCs, and the graft was either pre-incubated with or without MAX.16H5 IgG_1_ anti-human CD4 antibody. The survival in the group receiving a graft pre-incubated with MAX.16H5 IgG_1_ was significantly prolonged compared to the control group (*P* = 0.002, Log Rank test, Figure 1B). Data of WBC, lymphocyte, monocyte and granulocyte counts obtained from blood samples of mice is shown in Table 1. Blood sampling and parameter determination was performed throughout the whole experimental course and is presented as mean ± standard deviation (SD) per group (Table 1). The WBC counts in the control group decreased after transplantation and did not reach their initial values until the end of the experiment (Figure 1D, Table 1, not significant in Nemenyi test). The WBC counts in the group receiving a MAX.16H5 IgG_1_ pre-incubated graft deteriorated until day 20 and increased until the end of the experiment (Figure 1D, Table 1, day 20 vs. day −2, *P* = 0.003, Nemenyi test). The control animals showed decreased lymphocyte, monocyte, and granulocyte values until they reached termination criteria (Table 1, Figure S3). In the animals receiving a graft pre-incubated with MAX.16H5 IgG_1_, lymphocyte, monocyte, and granulocyte counts dropped initially but exceeded their initial values at the end of the experiment (Table 1, Figure S3).

### GVHD induction and immune cell reconstitution after transplantation of allogeneic bone marrow and spleen cells

Immune cell reconstitution after allogeneic transplantation of 1 × 10^7^ BMCs and 3 × 10^7^ splenocytes was investigated by flow cytometry of heparinized blood samples from recipient animals. In the experimental panel, the following markers were examined: murine CD3, murine CD4, human CD4, murine CD8, murine CD19, and HLA-DR3.

**Figure 2:**
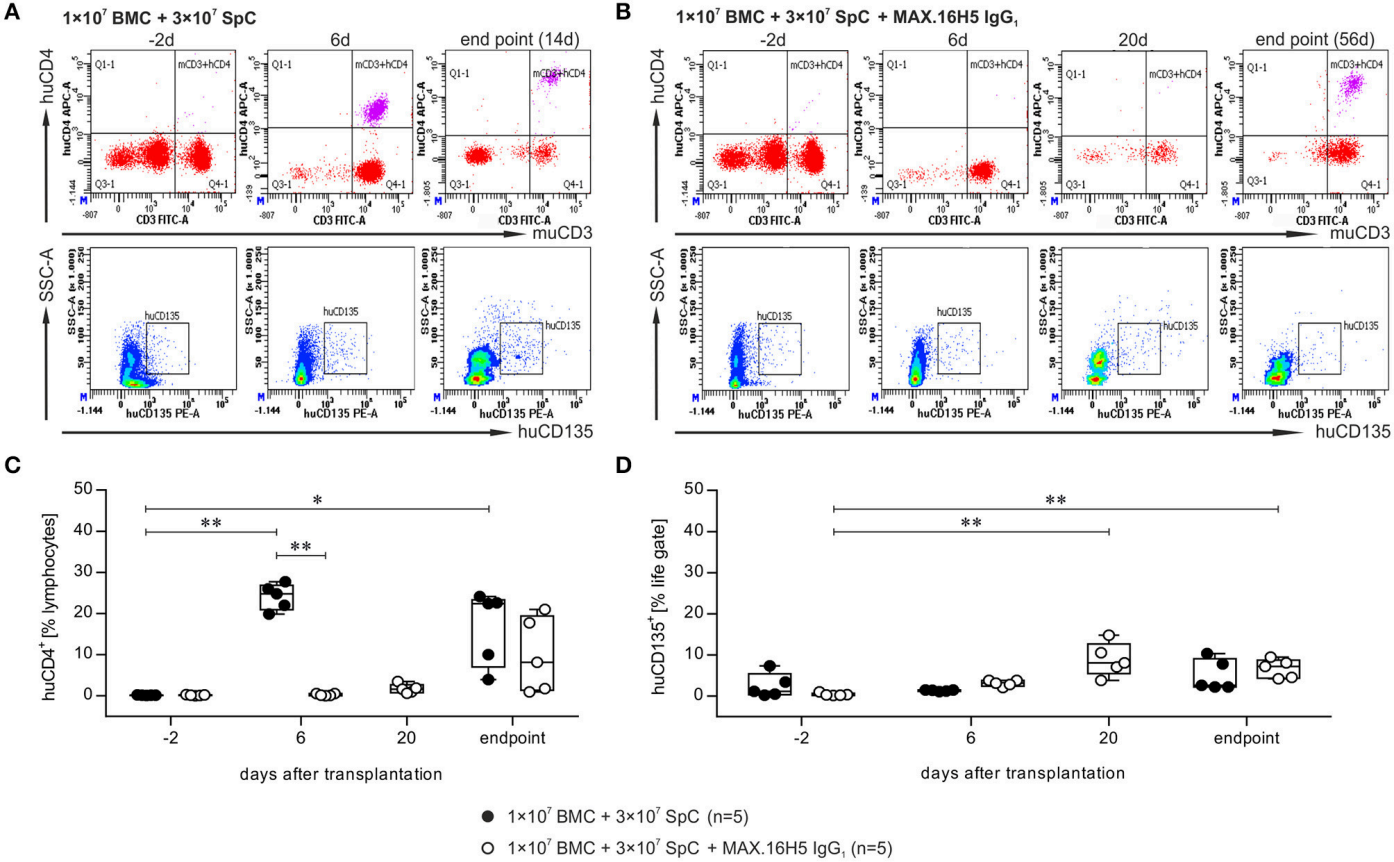
Flow cytometric analysis of the immune reconstitution (murine CD3^+^/human CD4^+^) as well as tumor growth (human CD135^+^) after transplantation of bone marrow and spleen cell grafts either pre-incubated with MAX.16H5 IgG_1_ or without antibody pre-treatment. All recipient animals received 1 × 10^7^ bone marrow cells (BMC) and 3 × 10^7^ splenocytes (SpC) in these experiments. **(A,B)** Representative plots obtained from flow cytometric analyses after transplantation of **(A)** one animal with untreated or **(B)** one animal with MAX.16H5 IgG_1_ pre-treated grafts at different time points (in **(A)**: day −2, day 6, endpoint; in **(B)**: day −2, day 6, day 20, endpoint) using specific antibodies against murine CD3/human CD4 or human CD135. In the lower rows, human CD135^+^ signals are depicted together with side-scattered light signals. **(C)** Human CD4^+^ events as percentages of lymphocyte gates in the blood samples of recipient mice. Every symbol indicates one animal at a defined time point. Engraftment and proliferation of human CD4^+^ cells were detected in five out of five animals receiving an untreated graft and in three out of five animals receiving the MAX.16H5 IgG_1_ pre-incubated grafts. The immune cell engraftment was delayed in animals receiving the MAX.16H5 IgG_1_ pre-incubated grafts. **(D)** Human CD135^+^ control staining of blood samples on labeled time points. **(C,D)** Nemenyi tests with Bonferroni correction computed the comparative statistics. *P < 0.05, **P < 0.01.

A detailed analysis was performed at four time points and exemplary scatter plots of flow cytometry measurements for one animal each receiving grafts with or without MAX.16H5 IgG_1_ pre-incubation are shown in Figures 2A,B and Figure S4. The data indicated as “endpoint” is a collection of measurements combining samples of mice, which were analyzed as soon as they had to be taken out of the experiment or as soon as they died which was not the exact same day in all cases. Detailed flow cytometry datasets of all analyzed mice are summarized in Table S1. Recipient animals, which received an untreated graft, showed a decreasing number of murine CD4^+^ events from 42.3 ± 3.2% on day −2 (*n* = 5/5) to 0.1 ± 0.2% on day 14 (*n* = 2/5) (Table S1A). In animals whose transplanted graft was pre-incubated with MAX.16H5 IgG_1_ murine CD4^+^ events dropped from 40.5 ± 3.9% on day −2 (*n* = 5/5) to 0.4 ± 0.4% on day 20 (*n* = 5/5) and to 1.1 ± 0.1% on day 56 (*n* = 2/5) (Table S1B), respectively. In the control animals, an increase in human CD4^+^ events from 0.2 ± 0.0% (day −2, *n* = 5/5) to 24.1 ± 3.1% (day 6, *n* = 5/5) and to 7.0 ± 4.3% (endpoint, day 14, *n* = 2/5) (Figures 2A,C and Table S1) was observed (day −2 vs. day 6, *P* = 0.003; day −2 vs. endpoint, *P* = 0.019; Nemenyi test). Animals receiving MAX.16H5 IgG_1_ pre-incubated grafts showed a delayed increase of human CD4^+^ events from 0.2 ± 0.1% (day −2, *n* = 5/5) and 0.3 ± 0.3% (day 6, *n* = 5/5) to 19.4 ± 2.3% (day 56, *n* = 2/5) (Figures 2B,C, Table S1, day 6 inter-group comparison, *P* = 0.003, Nemenyi test). On day 48, animals that received grafts pre-incubated with MAX.16H5 IgG_1_ showed a constant engraftment of human CD4^+^ cells (Table S1B). Additionally, increased numbers of CD8^+^ cytotoxic T cells were observed in both groups (Table S1). Only one mouse receiving a MAX.16H5 IgG_1_ pre-incubated graft showed constant murine CD19^+^ signals from day 20 after transplantation onwards (7.5%, Table S1B). Since no AML cells were co-transplanted in these experiments, similar percentages of human CD135^+^ events were detected in the blood from control animals transplanted with untreated grafts (5.0 ± 3.8%, *n* = 5/5, endpoint) and mice receiving MAX.16H5 IgG_1_ pre-incubated grafts (6.7 ± 2.3%, *n* = 5/5, endpoint) at the end of the experiment as expected (Figures 2A,B,D and Table S1). Still, an increase of CD135^+^ events toward the end of the experiment was observed in the group subjected to MAX.16H5 IgG_1_ pre-incubated transplants (day −2 vs. day 20, *P* = 0.001; day-2 vs. endpoint, *P* = 0.003; Nemenyi tests). Rank correlation tests (Kendall's tau) against the exact day after transplantation showed that CD135^+^ event rates increased in both groups with time (*r*_τ_ = 0.482, 95% confidence interval 0.057–0.907 without MAX.16H5 IgG_1_ pre-incubation; *r*_τ_ = 0.688, 95% confidence interval 0.505–0.870 with MAX.16H5 IgG_1_ pre-incubation). The confidence intervals might indicate a higher correlation in the MAX.16H5 IgG_1_ pre-treated group; however, possible between-group differences remain elusive.

### GVHD induction, immune cell reconstitution, and tumor growth after transplantation of allogeneic bone marrow and spleen cells in co-transplantation with 32D-FLT3^ITD^ cells

**Figure 3:**
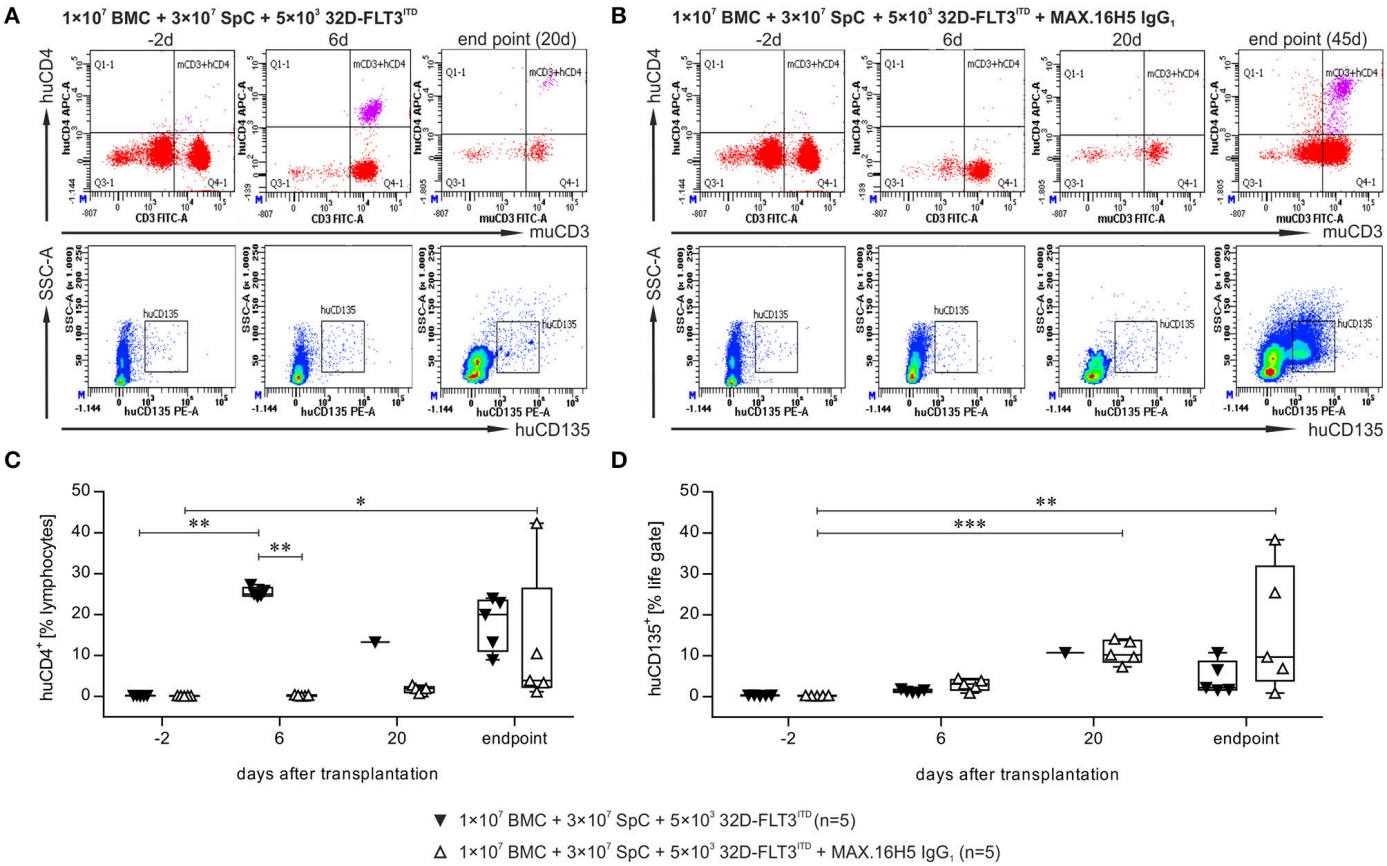
Flow cytometric analysis of the immune reconstitution (murine CD3^+^/human CD4^+^) as well as tumor growth (human CD135^+^) after transplantation of bone marrow and spleen cell grafts pre-incubated with MAX.16H5 IgG_1_ or without antibody pre-treatment in co-transplantation experiments with 5 × 10^3^ 32D-FLT3^ITD^ cells. All recipient animals received 1 × 10^7^ bone marrow cells (BMC) and 3 × 10^7^ splenocytes (SpC) in these experiments. **(A,B)** Representative plots of flow cytometric analyses after transplantation of **(A)** one animal with untreated or **(B)** one animal with MAX.16H5 IgG_1_ pre-treated grafts at different time points [in **(A)**: day −2, day 6, endpoint; in **(B)**: day −2, day 6, day 20, endpoint] using specific antibodies for murine CD3^+^/human CD4^+^ or human CD135^+^. In the lower rows, human CD135^+^ signals are depicted together with side-scattered light signals. **(C)** Human CD4^+^ events as percentages of lymphocyte gates in the blood samples of recipient mice. Every symbol indicates one animal at a defined time point. Engraftment and proliferation of human CD4^+^ cells were detected in five out of five animals receiving an antibody untreated graft and in two out of five animals receiving a MAX.16H5 IgG_1_ incubated graft. The immune cell engraftment was delayed in animals receiving the MAX.16H5 IgG_1_ pre-incubated grafts in comparison to the control group. **(D)** Engraftment of the 32D-FLT3^ITD^ cells was analyzed by immunofluorescent staining of blood samples (marker: human CD135). The human CD135^+^ counts in the blood samples from mice transplanted with 1 × 10^7^ BMCs + 3 × 10^7^ splenocytes + 5 × 10^3^ 32D-FLT3^ITD^ without MAX.16H5 IgG_1_ pre-treatment did not increase significantly until the end of the experiment (day 9–20) whereas the human CD135^+^ events in animals receiving a MAX.16H5 IgG_1_ pre-treated graft increased until they reached termination criteria (day 27–56). **(C,D)** Nemenyi tests with Bonferroni correction computed the comparative statistics. *P < 0.05, **P < 0.01, ***P < 0.001.

The flow cytometric analysis of blood samples of one representative animal collected from mice, which received a graft with or without pre-incubation with the antibody MAX.16H5 IgG_1_ in co-transplantation with 32D-FLT3^ITD^ cells, is shown in Figures 3A,B and Figure S5. A detailed analysis is shown at four time points (day −2, 6, 20 and endpoint). The data indicated as “endpoint” is a collection of measurements combining samples of mice, which were analyzed as soon as they reached termination criteria or as soon as they died which was not the exact same day in all cases. Detailed flow cytometry datasets of all analyzed animals are summarized in Table S2. Additionally, tumor growth was monitored by flow cytometric measurement of human CD135. The murine CD4^+^ counts in the blood samples from animals receiving an untreated graft decreased from 38.4 ± 7.9% (day −2, *n* = 5) to 0.2% (day 20, *n* = 1) and from 40.4 ± 3.8% (day −2, *n* = 5) to 4.2% (day 56, *n* = 1) in the groups receiving MAX.16H5 IgG_1_ pre-incubated grafts (Tables S2A,B). The engraftment of human CD4^+^ cells was delayed in those animals transplanted with MAX.16H5 IgG_1_ incubated grafts and 32D–FLT3^ITD^ AML cells (mean of human CD4^+^ events: 0.2 ± 0.0% (day −2, *n* = 5); 0.2 ± 0.1% (day 6, *n* = 5, inter-group comparison *P* = 0.006, Nemenyi test); 42.3% [endpoint, day 56, *n* = 1, day −2 vs. endpoint, *P* = 0.039, Nemenyi test)]. In the further course of the experiment however, CD4^+^ cell levels were higher than those of the corresponding control group [0.2 ± 0.0% (day −2, *n* = 5); 25.4 ± 1.2% (day 6, *n* = 5, day −2 vs. day 6, *P* = 0.003, Nemenyi test); 13.3% (day 20, *n* = 1)] (Figures 3A–C). The amount of CD8^+^ events was comparable in both groups (*n* = 5, Table S2). The CD19^+^ counts did not recover in the mice receiving an untreated graft (day 20: 0.6%, *n* = 1) such as in mice transplanted with an antibody treated graft (1.7 ± 1.5%, day 20, *n* = 5) (Table S2, Figure S5B). The 32D-FLT3^ITD^ tumor engraftment was detected by analysis of the blood samples for human CD135 using flow cytometry. The human CD135^+^ counts from mice transplanted with 1 × 10^7^ BMCs + 3 × 10^7^ splenocytes + 5 × 10^3^ 32D-FLT3^ITD^ without MAX.16H5 IgG_1_ pre-treatment did not significantly increase until the end of the experiment (4.6 ± 4.0%, *n* = 5, day 9–20) whereas the human CD135^+^ events in animals receiving a MAX.16H5 IgG_1_ pre-treated graft increased until the end of the experiment (17.9 ± 17.2%, *n* = 5, day 27–56; day −2 vs. day 20, *P* < 0.001; day −2 vs. endpoint, *P* = 0.008; Nemenyi tests) (Figure 3D and Table S2). However, additional rank correlation tests (Kendall's tau) against the precise day after transplantation indicated that CD135^+^ event ratios increased in both groups over time (*r*_τ_ = 0.842, 95% confidence interval 0.716–0.968 without MAX.16H5 IgG_1_ pre-incubation; *r*_τ_ = 0.665, 95% confidence interval 0.380–0.950 with MAX.16H5 IgG_1_ pre-incubation).

### Tumor growth in different organs after allogeneic transplantation

**Figure 4:**
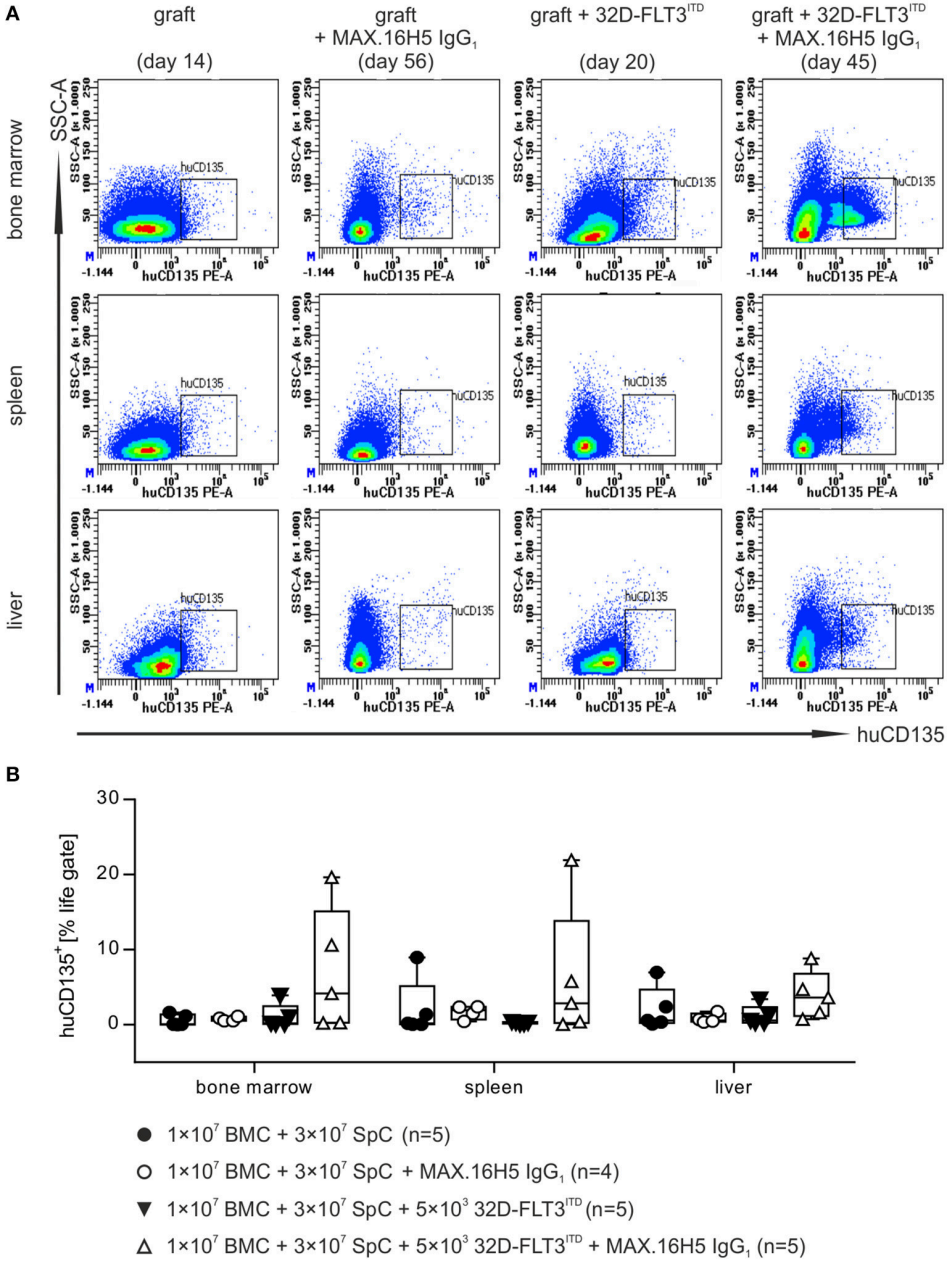
Flow cytometric analysis of human CD135^+^ events in different organs after transplantation of bone marrow and spleen cell grafts pre-incubated either with or without MAX.16H5 IgG_1_ in combination with or without co-transplantation of 32D-FLT3^ITD^ cells. The manifestation of human CD135^+^ cells in different organs (bone marrow, spleen and liver) from animals receiving 1 × 10^7^ bone marrow cells (BMC) and 3 × 10^7^ splenocytes (SpC) together with AML cells was analyzed by flow cytometry. **(A)** Representative plots from flow cytometric analyses of human CD135^+^ events after transplantation of untreated or MAX.16H5 IgG_1_-treated grafts with or without 5 × 10^3^ 32D-FLT3^ITD^ cells. At the endpoint, bone marrow, spleen, and liver samples from the transplanted mice were examined regarding human CD135 expression. **(B)** Human CD135^+^ events as percentage of the viable cell parent gate in organ preparations of recipient mice. Every symbol indicates one animal at a defined time point. The presented data is a collection of endpoint measurements combining samples from mice, which were analyzed as soon as they had to be taken out of the experiment or as soon as they died. Engraftment and proliferation of human CD135^+^ cells were detected in two out of five animals receiving MAX.16H5 IgG_1_ pre-incubated grafts in the co-transplantation experiments with 5 × 10^3^ 32D-FLT3^ITD^ cells. Kruskal-Wallis test indicated no significant differences (*P* = 0.171).

Bone marrow (BM), spleen, and liver samples were examined for tumor growth by using flow cytometric analysis of human CD135 at the endpoint of each animal. The presented data is a collection of endpoint measurements combining samples from mice, which were analyzed as soon as they had to be taken out of the experiment or as soon as they died. These endpoints ranged from day 8 (e.g., mice receiving an untreated graft) to day 56 (e.g., when mice received MAX.16H5 antibody incubated grafts). For mice receiving a treated or untreated graft (1 × 10^7^ BMCs + 3 × 10^7^ splenocytes) without co-transplantation of 32D-FLT3^ITD^ AML cells, human CD135^+^ events were not detectable neither in BM and liver nor in spleen preparations (Figures 4A,B and Table S3).

Mice receiving 32D-FLT3^ITD^ AML cells and an untreated graft showed no increased human CD135^+^ counts in the BM (1.1 ± 1.6%, *n* = 5), spleen (0.3 ± 0.1%, *n* = 5), or liver preparations (1.2 ± 1.3%, *n* = 5) (Figures 4A,B and Table S3). In comparison, human CD135^+^ signals were detected in BM, spleen, and liver preparations in two out of five mice receiving antibody pre-treated grafts and 32D-FLT3^ITD^ cells (BM: 7.0 ± 8.2%, spleen: 6.2 ± 9.1%, liver: 3.9 ± 3.2%, *n* = 5) (Figure 4 and Table S3).

### MAX.16H5 IgG_1_ pre-incubation reduces apoptotic cells in the gut (ileum) of transplanted mice

**Figure 5:**
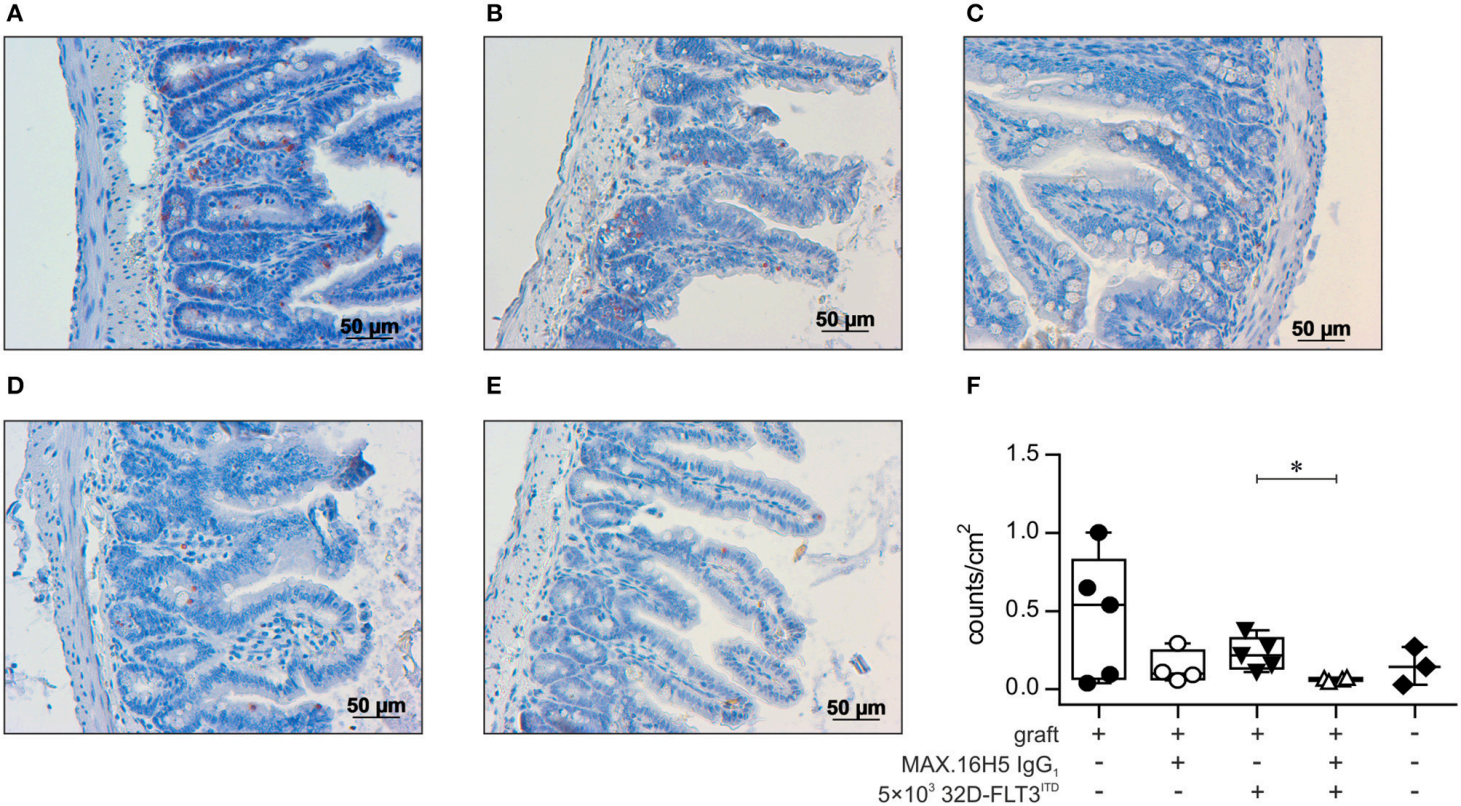
Histological analysis of gut tissue (ileum) from transplanted animals by TUNEL assay. Histological sections of gut tissues from all C3H/HeN mice receiving 1 × 10^7^ bone marrow cells (BMC) and 3 × 10^7^ splenocytes (SpC) after pre-incubation with MAX.16H5 IgG_1_ or an untreated graft with or without co-transplantation of 5 × 10^3^ 32D-FLT3^ITD^ cells were examined regarding apoptotic cell count. NaCl treated mice served as the control group. Five individual visual fields from one histological slide of the gut of each mouse were randomly chosen and microscopically recorded in 10 × magnification, and apoptotic cell counts were analyzed with ImageJ. **(A–E)** Representative histological ileum images of the transplanted animals (magnification 20 ×) after TUNEL staining. TUNEL stained gut sections of animals receiving 1 × 10^7^ bone marrow and 3 × 10^7^ spleen cells **(A)** without antibody pre-incubation and **(B)** with MAX.16H5 IgG_1_ pre-incubation are shown. **(C)** Ileum TUNEL staining from one mouse receiving a non-incubated graft together with 5 × 10^3^ 32D-FLT3^ITD^ cells. **(D)** Histological section from one mouse receiving a MAX.16H5 IgG_1_ pre-incubated graft together with 32D-FLT3^ITD^ AML cells. **(E)** TUNEL staining of a gut section from a NaCl control animal. Red areas indicate apoptotic nuclei in the basal crypts of the mucosa, which are a diagnostic criterion in acute GVHD (80). Apoptotic nuclei in the apical mucosal layer were excluded from the analysis. **(F)** Group-separated boxplots indicating the group medians and quartiles together with each symbol representing the mean of the examined apoptotic nuclei count per cm^2^ (based on the image in 10 × magnification) of all counted visual fields per mouse. Wilcoxon rank sum tests on unprocessed data were used for comparative statistics. *P < 0.05.

Apoptotic cells in the gut of mice were analyzed by TUNEL staining. Mice receiving an antibody untreated graft without co-transplantation of 32D-FLT3^ITD^ cells showed slightly higher apoptotic cell counts (median: 0.54 counts/cm^2^, 25% percentile: 0.07, 75% percentile: 0.83, *n* = 5, Figures 5A,F) compared to corresponding mice transplanted with a MAX.16H5 IgG_1_ antibody pre-incubated graft (median: 0.10 counts/cm^2^, 25% percentile: 0.06, 75% percentile: 0.25, *n* = 4) (Figures 5B,F). This difference was not significant (Wilcoxon rank sum test).

A significantly increased apoptosis rate (*P* = 0.016, Wilcoxon rank sum test) was detected in the gut from mice which were co-transplanted with 32D-FLT3^ITD^ cells and 1 × 10^7^ BMCs + 3 × 10^7^ splenocytes without prior MAX.16H5 IgG_1_ incubation (median: 0.22 counts/cm^2^, 25% percentile: 0.13, 75% percentile: 0.33, *n* = 5, Figures 5C,F) in comparison to mice receiving an antibody incubated graft and 32D-FLT3^ITD^ AML cells (median: 0.07 counts/cm^2^, 25% percentile: 0.05, 75% percentile: 0.07, *n* = 4, Figures 5D,F). Nevertheless, the apoptotic cell counts in the guts from the irradiated control group (median: 0.14 counts/cm^2^, 25% percentile: 0.03, 75% percentile: 0.27, *n* = 3) were comparable to those of the mice transplanted with 1 × 10^7^ BMCs, 3 × 10^7^ splenocytes and 5 × 10^3^ 32D-FLT3^ITD^ cells (Figures 5E,F). As only two comparisons were drawn, Wilcoxon rank sum tests on unprocessed data were performed without correction.

In the multifactorial analysis, MAX.16H5 IgG_1_ pre-treatment of the transplants was a significant (*P* = 0.002) as well as relevant factor indicating reduced clinical scores (interaction between the individual and time factor as cause of error variance, partial eta-squared = 0.450). On the other hand, neither the tumor transplantation nor the interaction between MAX.16H5 IgG_1_ pre-treatment and tumor transplantation exerted a significant influence. This indicates a worsened general health state of mice after the co-transplantation with AML cells carrying the FLT3-ITD mutation independently of the MAX.16H5 pre-incubation.

### Impact of graft transplantation on toxicity

For toxicity analysis, creatinine and uric acid concentrations in plasma samples of mice which received a graft (1 × 10^7^ bone marrow and 3 × 10^7^ spleen cells) after a short-term pre-incubation with MAX.16H5 IgG_1_ or without pre-incubation in combination with 5 × 10^3^ 32D-FLT3^ITD^ cells were examined (For detailed description of the performed assays see also Supplementary Materials and Methods). A strong and significant increase in creatinine levels was detected in both groups starting on day 6 after transplantation [without antibody: day−2: 179.60 ± 11.08 μmol/L, day 6: 566.40 ± 46.85 μmol/L, *P* < 0.001 (*n* = 5); with anti-human CD4 antibody MAX.16H5 IgG_1_: day−2: 226.8 ± 154.37 μmol/L, day 6: 639.60 ± 45.59 μmol/L, *P* < 0.001 (*n* = 5)] until the end of the experiment (Figure S6A, with anti-human CD4 antibody MAX.16H5 IgG_1_: day −2 vs. day 14, vs. day 20, and vs. day 28, *P* < 0.001, pre-defined contrasts only taking minimum sample sizes of 4 into account in simultaneous tests for general linear hypothesis, Bonferroni adjustment). The plasma samples of mice in the group receiving an untreated graft showed significantly higher uric acid concentration on day 6 (305.94 ± 39.38 μmol/L, *n* = 5) than on day −2 (137.48 ± 30.81 μmol/L, *P* < 0.001, pre-defined contrasts only taking minimum sample sizes of 4 into account in simultaneous tests for general linear hypothesis, Bonferroni adjustment). In comparison, no difference in the uric acid concentration was observed between day −2 and day 6 in the plasma of those mice receiving MAX.16H5 IgG_1_ pre-treated grafts (Figure S6B). The uric acid concentrations measured on day 6 were significantly different between the animals receiving grafts either without (305.94 ± 39.38 μmol/L, *n* = 5) or with MAX.16H5 IgG_1_ pre-incubation (156.84 ± 41.12 μmol/L, *n* = 5, *P* = 0.003) (Figure S6B). Nevertheless, starting on day 14 increased uric acid concentration were observed within the group receiving an antibody pre-incubated graft (day−2: 120.93 ± 17.61 μmol/L, *n* = 5, day 14: 215.84 ± 97.52 mol/L, *n* = 5). Uric acid concentrations in plasma samples of these mice receiving an antibody pre-incubated graft were significantly different between day−2 (120.93 ± 17.61 μmol/L, *n* = 5) and day 28 (254.30 ± 59.14 μmol/L, *n* = 5, *P* = 0.019) and were maintained until the end of the experiment (day 56: 390.70 μmol/L, *n* = 1). Calcium and phosphate plasma levels were not significantly different between mice receiving grafts with or without MAX.16H5 IgG_1_ antibody pre-incubation (data not shown).

## Discussion

GVHD is one of the main complications leading to a high mortality rate among patients after allogenic HSCT (57–59). AML patients presenting with an aberrant FLT genotype are regularly transplanted when their prognosis is intermediate or poor (60). These patients receive extensive chemotherapy and midostaurin as induction therapy prior to HSCT (60). To date, HSCT is often the only curative therapy option for patients suffering from leukemia (61, 62). New therapy strategies need to be designed ensuring the prevention of GVHD while supporting or maintaining the GVL effect (63, 64). In the past, it was shown that the graft treatment with the anti-human CD4 antibodies MAX.16H5 IgG_1_ (murine) or IgG_4_ (chimeric) was able to prevent GVHD in a xenogeneic NSG HSCT model (27) and also to preserve the GVL effect in a murine mastocytoma model (MAX.16H5 IgG_1_) (26). Due to these promising results, a murine full MHC mismatch transplantation model for AML was established to investigate whether MAX.16H5 IgG_1_ also exerts a beneficial effect regarding GVHD prevention in the background of leukemia. A highly aggressive AML subtype carrying the ITD mutation in the FLT3 receptor was used in this tumor model (65). The oncogenic potential of 32D cells transfected with FLT3^ITD^ or FLT3^wt^ has already been described in a syngeneic setting elsewhere (45). It is important to note that wild-type FLT3 is regularly expressed on CD34^+^ hematopoietic stem cells and plays a role in the development of different immune cells (e.g., NK cells, dendritic cells, B cells) [reviewed in (66)]. For co-transplantation experiments, 32D-FLT3^ITD^ cells were administered together with the allogeneic hematopoietic graft of CD4/DR3 mice, which was shortly pre-incubated with MAX.16H5 IgG_1_ before transplantation in C3H/HeN mice as previously described (26). In this work it could be shown that lethally irradiated mice, which received BMC and splenocyte transplants from CD4/DR3 mice without MAX.16H5 IgG_1_ pre-treatment died within 20 days due to the development of GVHD. In comparison, the groups transplanted with MAX.16H5 IgG_1_ pre-treated grafts survived significantly longer in this model. We assume that the development of severe GVHD was hampered due to the antibody treatment. Nevertheless, the appearance of severe acute GVHD was not prevented in all mice receiving antibody pre-treated grafts, but the GVHD development was decelerated and effectively reduced in this transplantation setting. It should be pointed out that the development of GVHD in mice models is influenced by different parameters such as the applied irradiation dose, a variable proportion of T cell subsets (e.g., CD4^+^) residing in the graft, and genetic disparity in MHC and minor histocompatibility antigens between donor and recipient [reviewed in (67)].

The engraftment of the transplanted immune cell grafts was confirmed by flow cytometric detection of circulating human CD4^+^ events in the blood of recipient animals. Within a week following transplantation, no T cells positive for human CD4 were detected in those groups receiving grafts pre-incubated with MAX.16H5 IgG_1_ antibodies (with or without co-transplanted AML cells). In an earlier study it was shown that the engraftment of MAX.16H5 antibody incubated grafts was delayed in comparison to mice receiving a graft treated with an isotype control antibody (27). Eventually, by the end of the experiments, 5 out of 6 mice showed a stable engraftment of human immune cells (27). The appearance of murine CD3^+^/murine CD8^+^ events in the blood samples was comparable between the groups receiving grafts with or without antibody pre-treatment and with or without AML co-administration. It is known that the clinical outcome after HSCT is strongly dependent on the cellular composition of the donor grafts (68). In several studies, different researchers showed that the depletion of T cells and a non-myeloablative conditioning regimen led to a higher risk of graft failure and decreased graft function in transplanted patients (69). We already showed in previous *in vitro* experiments that in comparison to the depleting antibody OKT3 the MAX.16H5 IgG_1_ antibody was not able to deplete T cells by activating the complement system (complement-dependent cytotoxicity) (27). We also discussed a potential mode of action for the induced tolerance in regard to transplanted stem cell grafts after MAX.16H5 IgG_1_ antibody incubation elsewhere (27, 29). The binding of the antibody to CD4^+^ T cells may shift their development toward an anergic, tolerogenic state resulting in a decreased GVHD activation after HSCT (27, 29). Tolerance induction by a CD4 directed antibody was also described by Helling et al. where the humanized CD4-specific monoclonal antibody tregalizumab was examined regarding its immunomodulatory potential (70). Tregalizumab modulated regulatory T cells in a T cell receptor-independent manner inducing an unique phosphorylation pattern of signaling molecules in CD4^+^ cells (70). The antibody recognizes the second domain of the CD4 molecule on T cells (70). In contrast, the binding of CD4-directed antibodies to the first domain D1 of CD4 was described to be possibly connected to the induction of T cell anergy due to a steric hindrance of the CD4/MHC class II interaction after antibody binding (70). However, the mechanisms underlying the immunomodulatory features of the MAX.16H5 IgG_1_ antibody still remain elusive and have to be addressed in further studies.

According to data obtained in a former study (29), we showed in this work that a single dose treatment with MAX.16H5 IgG_1_ did not cause graft failure and was sufficient to prevent GVHD in allogeneic transplanted mice. Nevertheless, in the present study two out of five mice, which received antibody pre-incubated grafts, showed increased counts of human CD135^+^ events toward the end of the experiment, thus indicating a higher tumor burden and engraftment of the FLT3^ITD^ AML cells. It has to be noted that mice receiving an antibody-incubated graft survived significantly longer than mice, which were transplanted with control grafts, leading to different experimental “endpoints” in this setting. The prolonged life span of these particular mice may cause re-proliferation of 32D-FLT3^ITD^ AML explaining the increased CD135^+^ events. However, at comparable time points (until day 20), both groups (with and without MAX.16H5 antibody incubation) showed similar levels of CD135^+^ events in the blood. The growth and kinetics of tumor cells (e.g., AML) in mouse models are of special importance for dataset analysis and interpretation. Therefore, extensive literature search was implemented beforehand regarding AML tumorigenesis in mouse models. In these studies, AML cells were mainly transplanted in a syngeneic setting without irradiation of the animals. Mizuki et al. investigated 32D-FLT3^ITD^ and 32D-FLT3^wt^ cells regarding their tumorigenic potential and the development of leukemia-resembling disease in a syngeneic setting using non-irradiated C3H mice (45). The group showed that the injection of 1 × 10^6^ 32D-FLT3^ITD^ cells led to a rapid development of a leukemia-like disease and that the mice died within 4–5 weeks after tumor cell application. The animals also showed signs of splenomegaly. In contrast, mice receiving 32D-FLT3^wt^ cells did not develop a leukemic disease until up to 3 months after tumor cell injection (45). According to these data, we showed in a previous work that non-irradiated C3H mice transplanted with 1 × 10^6^ 32D-FLT3^ITD^ cells (syngeneic setting) died within 21 days after tumor cell application (71). Moreover, an increased CD135^+^ cell count was detected by flow cytometry in peripheral blood samples of mice which died a few days after blood collection. In preparation for the immune cell graft transplantation used in this current study, irradiation was performed in all animals to eradicate their immune system. The comparison between models described in literature and the allogeneic transplantation setting we describe in this manuscript is hampered due to the lethal irradiation of mice. Irradiated mice will die when hematopoiesis is not rescued by the transplantation of an immune cell graft. Although we impressively showed that GVHD was significantly reduced in our co-transplantation experiments and the antibody did not cause graft failure, our FLT3^ITD^ positive AML C3H mouse model revealed some limitations, especially regarding the AML kinetics due to the lethal irradiation protocol. Further investigations are required to obtain data regarding GVHD and GVL activity of MAX.16H5 antibody-incubated immune cell grafts using an NSG xenograft transplantation model in the background of AML. Using this approach, mice do not have to be lethally irradiated in preparation for graft transplantation and the kinetics of the tumor cell growth can be implemented additionally.

One of the examined mice receiving 32D-FLT3^ITD^ AML cells and the pre-incubated graft showed a lower amount of CD135^+^ events but an increased count for CD8^+^ events, whereas the results were reversed in a second mouse. Since it is known that CD8^+^ cells play a major role in the immune defense against tumor cells [reviewed in 72)], there may also be a correlation between tumor cell counts and CD8^+^ cell counts in this experimental setting. A plausible deduction of a specific mechanism and a conclusion for a correlation was not made since the effects were observed in single animals only. For a better understanding of the underlying mechanism further studies have to focus specifically on this topic. Regarding our used assay we cannot exclude a cross-reactivity of the used mouse anti-human CD135 antibody since low levels of human CD135^+^ events were also detected in mice that were not co-transplanted with AML cells.

However, one mouse of the group receiving the antibody pre-incubated graft and FLT3^ITD^ AML cells survived without developing AML and showed a successful engraftment without any signs of GVHD. We assume that mice receiving an antibody treated graft together with AML cells exhibited a prevention of GVHD by the graft incubation with the anti-human CD4 antibody MAX.16H5 IgG_1_. Unfortunately, the GVL effect mediated by the graft was not strong enough and HSCT alone was not sufficient to cure AML carrying a FLT3^ITD^ mutation in this model. In general, co-transplantation of 32D-FLT3^ITD^ (mutated) AML cells with an immune cell graft in lethally irradiated mice revealed that MAX.16H5 IgG_1_ antibody pre-incubation of the graft cells prolonged their survival significantly. Nevertheless, when 32D-FLT3^wt^ AML cells were used for co-transplantation, lower splenocyte counts were sufficient to reduce AML cells with or without antibody pre-incubation of the graft reflecting the aggressive proliferation properties of FLT3^ITD^ AML cells. Moreover, in comparison to the control group (without MAX.16H5 IgG_1_ pre-incubation), no tumor cells were detected in the blood of the transplanted mice on day 54 after transplantation if mice received an antibody treated graft together with 32D-FLT3^wt^ AML cells. When ITD-mutated AML cells were investigated under the same experimental conditions, the control group receiving an untreated graft died within 18 days, whereas the mice receiving a MAX.16H5 IgG_1_ treated graft died within 35 days after allogeneic transplantation. A slight increase in human CD135^+^ counts was observed in both groups on day 5, possibly due to a subsequent manifestation of the tumor cells in the organs. Because of this tissue manifestation, no AML cells were detectable in the periphery. Even though a long-term anti-tumor effect was not mediated by graft transplantation, the incubation with the anti-human CD4 antibody MAX.16H5 IgG_1_ improved the survival of the mice significantly. The efficient prevention of GVHD after immune cell transplantation mediated by the MAX.16H5 antibody graft incubation prolonged the survival of the respective animals paving the way for 32D-FLT3^ITD^ AML cell proliferation later on thereby causing the death of all mice examined. Unfortunately, the transplantation of higher graft cell counts in combination with MAX.16H5 antibody pre-incubation only further prolonged the survival of the mice but showed no long lasting anti-tumor effect toward the ITD mutated AML cells in this model. These findings are supported by the work of other groups, showing that constitutively activated STAT5 in FLT3^ITD^ mutated cells leads to uncontrolled proliferation, factor independent growth and radiation resistance in humans and mice (45).

AML patients carrying cytogenetically mutated FLT3^ITD^ are affected by high post-HSCT relapse rates and diminished overall survival (73–75). A study from Whiteman et al. showed that 87% of patients carrying a FLT3^ITD/−^ AML died within 12 months. In comparison, 74% of patients with a wild type (FLT3^wt/wt^) AML and 65% of patients with a heterozygous (FLT3^ITD/wt^) AML were alive 12 months after autologous transplantation (36). Another study by Bienz et al. revealed a three-year overall survival of one patient with FLT3^ITD^ AML (out of 19 patients) compared to 11 patients with FLT3^wt^ AML (out of 48 patients) after intensive chemotherapy and allogeneic transplantation (76). Comparable results were reviewed by Beitinjaneh et al. in an article where 11 different studies were examined (77). This indicates that the cure of FLT3^ITD^ mutated AML by allogeneic HSCT can be challenging, since this mutation is involved in the activation of proliferative signaling cascades inhibiting hematopoietic differentiation and apoptosis in these cells [reviewed in (78)]. However, HSCT and the graft-mediated GVL effect are key players in the successful treatment of many AML patients (79).

The results of the present study indicate that the anti-human CD4 antibody MAX.16H5 IgG_1_ successfully impairs GVHD development following allogeneic HSCT which is still the main complication arising from HSCT. Although the successful allogeneic transplantation did not induce long-term anti-tumor effects in the mice, the prevention of GVHD by incubating graft cells with the anti-CD4 antibody prolonged their survival significantly thereby opening a time frame for additional treatment options. In further experiments, the results regarding FLT3^ITD^ mutated AML will be investigated in NSG mice in order to verify our findings in a more patient-related setting.

## Author contributions

NH, SF, and GB designed the research; NH, CM, AM, CH, CW, and SF ran the experiments; NH, CM, AK, SF, LS, SD, DG, AW, and CH analyzed results; NH, CM, AK, BZ, LS, SF, and CH created figures and wrote the paper.

### Conflict of interest statement

The authors declare that the research was conducted in the absence of any commercial or financial relationships that could be construed as a potential conflict of interest.

## Abbreviations

AML: acute myeloid leukemia
BM: bone marrow
BMC: bone marrow cells
CD: cluster of differentiation
CTL: cytotoxic T lymphocyte
FLT3: FMS like tyrosine-protein kinase 3
GVHD: graft-vs. -host disease
GVL: graft-vs.-leukemia
HLA-DR3: human leukocyte antigen isotype DR3
HSCT: hematopoietic stem cell transplantation
hu: human
ITD: internal tandem duplication
MHC: major histocompatibility complex
mu: murine
NK: natural killer
NSG: NOD.Cg-Prkdc^scid^ IL-2rg^tm1Wjl^/SzJ
PBS: phosphate buffered saline
SD: standard deviation
SpC: splenocytes
SSC-A: side scatter area
TUNEL: TdT-mediated dUTP-biotin nick end labeling
WBC: white blood cell
wt: wild type.
