# Supplementary material for: Incubation of Immune Cell Grafts With MAX.16H5 IgG1 Anti-Human CD4 Antibody Prolonged Survival After Hematopoietic Stem Cell Transplantation in a Mouse Model for Fms Like Tyrosine Kinase 3 Positive Acute Myeloid Leukemia
Source: Front Immunol. 2018 Oct 22;9:2408. doi: 10.3389/fimmu.2018.02408 (PMC6204383; doi:10.3389/fimmu.2018.02408)
Supplement: Supplementary file 1 [file Data_Sheet_1.docx]

Supplementary Material

Incubation of Immune Cell Grafts with MAX.16H5 IgG_1_ Anti-Human CD4 Antibody Prolonged Survival after Hematopoietic Stem Cell Transplantation in a Mouse Model for Fms Like Tyrosine Kinase 3 Positive Acute Myeloid Leukemia

**Nadja Hilger^1,2+^*, Claudia Mueller^1+^, Lilly, Stahl^1^, Anne M. Mueller^1^, Bianca Zoennchen^1^, Sarah Dluczek^1^, Christoph Halbich^1^, Claudia Wickenhauser^3^, Dennis Gerloff^4^, Alexander A. Wurm^5^, Gerhard Behre^5^, Anna Kretschmer^1#^, and Stephan Fricke^1#^**

*** Correspondence:** Nadja Hilger: nadja.hilger@izi.fraunhofer.de

# Supplementary tables and figures

## Supplementary tables

**Table S1.** Flow cytometric analysis of blood samples collected from mice receiving 1×10^7^ bone marrow cells (BMC) and 3×10^7^ spleen cells (SpC) either **(A)** without MAX.16H5 IgG_1_ pre-incubation (* = mean **±** SD day 14,** = mean **±** SD all animals) or **(B)** with antibody incubation (* = mean **±** SD day 56,** = mean **±** SD all animals) in transplantation experiments. Each column of the tables represents one single recipient animal (#1–#5).

| **A** |  |  |  |  |  |  |  |  |  |  |  | |  | |  | |  | |  | |  | |  | |  |  |  |  |  |  |  |
| --- | --- | --- | --- | --- | --- | --- | --- | --- | --- | --- | --- | --- | --- | --- | --- | --- | --- | --- | --- | --- | --- | --- | --- | --- | --- | --- | --- | --- | --- | --- | --- |
|  | **day -2** | | | | | | **day 6** | | | | | | | **endpoint** | | | | | | | | | | | |  |  |  |  |  |  |
| **mouse** | **#1** | **#2** | **#3** | **#4** | **#5** | **mean ± SD** | **#1** | **#2** | **#3** | **#4** | **#5** | **mean ± SD** | | **#1**  **(8d)** | | **#2**  **14d)** | | **#3**  **(8d)** | | **#4**  **(14d)** | | **#5**  **(9d)** | | **mean ± SD** | |  |  |  |  |  |  |
| %muCD3/muCD4 | 43.3 | 36.7 | 43.5 | 44.7 | 43.1 | 42.3 ± 3.2 | 0.8 | 0.9 | 1.3 | 0.6 | 1.1 | 0.9 ± 0.3 | | 0.3 | | 0.3 | | 0.4 | | 0.0 | | 0.8 | | 0.1 ± 0.2* | |  |  |  |  |  |  |
| %muCD3/huCD4 | 0.1 | 0.2 | 0.1 | 0.2 | 0.2 | 0.2 ± 0.0 | 19.9 | 26.0 | 24.8 | 22.0 | 27.7 | 24.1 ± 3.1 | | 22.4 | | 10.0 | | 22.6 | | 4.0 | | 24.1 | | 7.0 ± 4.3* | |  |  |  |  |  |  |
| %muCD3/muCD8 | 15.4 | 11.9 | 15.1 | 14.1 | 13.8 | 14.1 ± 1.4 | 74.5 | 68.3 | 68.3 | 73.7 | 67.9 | 70.5 ± 3.2 | | 61.0 | | 14.7 | | 63.3 | | 7.1 | | 55.5 | | 10.9 ± 5.4* | |  |  |  |  |  |  |
| %muCD19 | 29.0 | 33.4 | 26.5 | 29.2 | 31.1 | 29.9 ± 2.6 | 0.4 | 0.3 | 0.6 | 0.2 | 0.3 | 0.4 ± 0.1 | | 0.0 | | 0.2 | | 0.1 | | 0.2 | | 0.6 | | 0.2 ± 0.0* | |  |  |  |  |  |  |
| %huCD135 | 7.4 | 1.1 | 3.4 | 0.2 | 0.5 | 2.5 ± 3.0 | 1.1 | 1.5 | 1.4 | 1.5 | 1.2 | 1.3 ± 0.2 | | 2.6 | | 10.3 | | 2.3 | | 7.8 | | 2.2 | | 9.1 ± 1.8*  5.0 ± 3.8** | |  |  |  |  |  |  |
|  |  |  |  |  |  |  |  |  |  |  |  | |  | |  | |  | |  | |  | |  | |  |  |  |  |  |  |  |
| **B** |  |  |  |  |  |  |  |  |  |  |  | |  | |  | |  | |  | |  | |  | |  |  |  |  |  |  |  |
|  | **day -2** | | | | | | **day 6** | | | | | | | **day 20** | | | | | | | | | | | | **endpoint** | | | | | |
| **mouse** | **#1** | **#2** | **#3** | **#4** | **#5** | **mean ± SD** | **#1** | **#2** | **#3** | **#4** | **#5** | **mean ± SD** | | **#1** | | **#2** | | **#3** | | **#4** | | **#5** | | **mean ± SD** | | **#1**  **(42d)** | **#2**  **(20d)** | **#3**  **(56d)** | **#4**  **(48d)** | **#5**  **(56d)** | **mean ± SD** |
| %muCD3/muCD4 | 35.4 | 39.5 | 46.0 | 41.9 | 39.5 | 40.5 ± 3.9 | 2.3 | 0.8 | 0.9 | 1.6 | 2.6 | 1.7 ± 0.8 | | 1.1 | | 0.2 | | 0.4 | | 0.0 | | 0.2 | | 0.4 ± 0.4 | | 2.2 | 0.2 | 1.1 | 0.4 | 1.2 | 1.1 ± 0.1* |
| %muCD3/huCD4 | 0.1 | 0.1 | 0.3 | 0.2 | 0.2 | 0.2 ± 0.1 | 0.6 | 0.6 | 0.1 | 0.0 | 0.1 | 0.3 ± 0.3 | | 3.5 | | 1.0 | | 1.9 | | 1.6 | | 0.6 | | 1.7 ± 1.1 | | 1.8 | 1.0 | 17.8 | 8.1 | 21.0 | 19.4 ± 2.3* |
| %muCD3/muCD8 | 11.6 | 13.3 | 15.7 | 14.7 | 13.5 | 13.8 ± 1.6 | 82.5 | 87.4 | 84.9 | 83.6 | 87.1 | 85.1 ± 2.2 | | 29.1 | | 44.7 | | 42.3 | | 60.4 | | 58.5 | | 47.0 ± 12.8 | | 61.4 | 44.7 | 22.5 | 40.2 | 60.1 | 41.3 ± 26.6* |
| %muCD19 | 21.2 | 26.0 | 27.3 | 27.3 | 23.7 | 25.1 ± 2.6 | 0.1 | 0.2 | 0.7 | 0.2 | 0.4 | 0.3 ± 0.2 | | 0.3 | | 1.0 | | 7.5 | | 2.3 | | 1.4 | | 2.5 ± 2.9 | | 0.7 | 1.0 | 47.4 | 3.0 | 0.7 | 24.0 ± 33.0* |
| %huCD135 | 1.0 | 0.4 | 0.2 | 0.2 | 0.3 | 0.4 ± 0.3 | 3.8 | 2.1 | 3.8 | 3.2 | 2.8 | 3.1 ± 0.7 | | 7.1 | | 8.1 | | 3.8 | | 14.8 | | 10.5 | | 8.9 ± 4.1 | | 4.2 | 8.1 | 9.5 | 7.2 | 4.5 | 7.0 ± 3.5*  6.7 ± 2.3** |

**Table S2.** Flow cytometric analysis of blood samples collected from mice receiving 1×10^7^ bone marrow cells (BMC) and 3×10^7^ spleen cells (SpC) together with 5×10^3^ 32D-FLT3^ITD^ cells either **(A)** without antibody incubation or **(B)** with MAX.16H5 IgG_1_ pre-incubation in transplantation experiments. Each column of the tables represents one single recipient animal (#1–#5).

| **A** |  |  |  |  |  |  |  |  |  |  |  |  |  |  |  |  |  |  |  |  |  |  |  |  |
| --- | --- | --- | --- | --- | --- | --- | --- | --- | --- | --- | --- | --- | --- | --- | --- | --- | --- | --- | --- | --- | --- | --- | --- | --- |
|  | **day -2** | | | | | | **day 6** | | | | | | **day 20** | | | | | | **endpoint** | | | | | |
| **mouse** | **#1** | **#2** | **#3** | **#4** | **#5** | **mean ± SD** | **#1** | **#2** | **#3** | **#4** | **#5** | **mean ± SD** | **#1** | **#2** | **#3** | **#4** | **#5** | **mean ± SD** | **#1**  **(9d)** | **#2**  **(20d)** | **#3**  **(9d)** | **#4**  **(14d)** | **#5**  **(9d)** | **mean ± SD** |
| %muCD3/muCD4 | 35.0 | 42.1 | 42.7 | 45.9 | 26.1 | 38.4 ± 7.9 | 0.9 | 1.1 | 1.1 | 0.8 | 0.8 | 0.9 ± 0.2 | - | 0.2 | - | - | - | 0.2 | 0.0 | 0.2 | 0.4 | 0.2 | 0.6 | 0.3 ± 0.2 |
| %muCD3/huCD4 | 0.2 | 0.2 | 0.2 | 0.2 | 0.1 | 0.2 ± 0.0 | 25.8 | 27.4 | 24.6 | 25.0 | 24.4 | 25.4 ± 1.2 | - | 13.3 | - | - | - | 13.3 | 9.0 | 13.3 | 20.0 | 22.9 | 24.0 | 17.8 ± 6.5 |
| %muCD3/muCD8 | 12.4 | 14.1 | 14.1 | 15.3 | 5.1 | 12.2 ± 4.1 | 65.9 | 65.4 | 63.6 | 68.1 | 57.1 | 64.0 ± 4.2 | - | 50.0 | - | - | - | 50.0 | 10.9 | 50.0 | 42.2 | 43.0 | 47.2 | 38.7 ± 15.8 |
| %muCD19 | 35.4 | 30.7 | 25.9 | 24.3 | 12.3 | 25.7 ± 8.7 | 0.9 | 0.4 | 0.3 | 0.5 | 0.8 | 0.6 ± 0.3 | - | 0.6 | - | - | - | 0.6 | 0.0 | 0.6 | 0.1 | 1.3 | 0.5 | 0.5 ± 0.5 |
| %huCD135 | 0.4 | 0.3 | 0.4 | 0.2 | 0.3 | 0.3 ± 0.1 | 1.6 | 1.9 | 1.0 | 1.1 | 1.3 | 1.4 ± 0.4 | - | 10.7 | - | - | - | 10.7 | 2.2 | 10.7 | 1.6 | 6.6 | 1.8 | 4.6 ± 4.0 |
|  |  |  |  |  |  |  |  |  |  |  |  |  |  |  |  |  |  |  |  |  |  |  |  |  |
| **B** |  |  |  |  |  |  |  |  |  |  |  |  |  |  |  |  |  |  |  |  |  |  |  |  |
|  | **day -2** | | | | | | **day 6** | | | | | | **day 20** | | | | | | **endpoint** | | | | | |
| **mouse** | **#1** | **#2** | **#3** | **#4** | **#5** | **mean ± SD** | **#1** | **#2** | **#3** | **#4** | **#5** | **mean ± SD** | **#1** | **#2** | **#3** | **#4** | **#5** | **mean ± SD** | **#1**  **(34d)** | **#2**  **(56d)** | **#3**  **(27d)** | **#4**  **(28d)** | **#5**  **(45d)** | **mean ± SD** |
| %muCD3/muCD4 | 40.7 | 34.1 | 44.4 | 41.6 | 40.9 | 40.4 ± 3.8 | 0.9 | 3.7 | 1.5 | 1.6 | 1.7 | 1.9 ± 1.1 | 0.6 | 1.0 | 0.7 | 0.2 | 0.9 | 0.7 ± 0.3 | 2.6 | 4.2 | - | 0.6 | 0.5 | 2.0 ± 1.8 |
| %muCD3/huCD4 | 0.2 | 0.2 | 0.1 | 0.1 | 0.1 | 0.2 ± 0.0 | 0.4 | 0.1 | 0.1 | 0.3 | 0.2 | 0.2 ± 0.1 | 2.8 | 1.9 | 1.2 | 0.8 | 2.0 | 1.7 ± 0.8 | 3.9 | 42.3 | - | 3.4 | 10.5 | 15.0 ± 18.5 |
| %muCD3/muCD8 | 15.6 | 12.5 | 14.6 | 12.9 | 14.0 | 13.9 ± 1.3 | 86.3 | 55.6 | 78.6 | 87.4 | 82.4 | 78.1 ± 13.0 | 53.1 | 56.2 | 53.3 | 37.7 | 63.9 | 52.8 ± 9.5 | 24.8 | 17.2 | - | 71.1 | 52.4 | 41.4 ± 24.9 |
| %muCD19 | 31.1 | 38.3 | 26.4 | 32.4 | 33.3 | 32.3 ± 4.3 | 0.9 | 1.2 | 0.4 | 0.4 | 0.6 | 0.7 ± 0.3 | 1.8 | 0.0 | 4.0 | 0.8 | 1.7 | 1.7 ± 1.5 | 2.6 | 10.6 | - | 0.6 | 0.6 | 3.6 ± 4.8 |
| %huCD135 | 0.1 | 0.2 | 0.3 | 0.2 | 0.3 | 0.2 ± 0.1 | 2.4 | 3.9 | 4.4 | 3.1 | 0.9 | 2.9 ± 1.4 | 7.3 | 13.4 | 9.7 | 14.1 | 10.2 | 10.9 ± 2.8 | 38.3 | 0.9 | - | 6.9 | 25.4 | 17.9 ± 17.2 |

**Table S3.** Flow cytometry data of human CD135 expression (human CD135^+^events [% life gate]) in BM, spleen and liver of mice after graft transplantation. Analyses were performed after completion of the experimental course. Each column of the tables represents one single recipient animal (#1–#5). BMC = bone marrow cells, SpC = spleen cells

|  | **bone marrow** | | | | | | **spleen** | | | | | | **liver** | | | | | |
| --- | --- | --- | --- | --- | --- | --- | --- | --- | --- | --- | --- | --- | --- | --- | --- | --- | --- | --- |
| **mouse** | **#1** | **#2** | **#3** | **#4** | **#5** | **mean ± SD** | **#1** | **#2** | **#3** | **#4** | **#5** | **mean ± SD** | **#1** | **#2** | **#3** | **#4** | **#5** | **mean ± SD** |
| 1×10^7^ BMC + 3×10^7^ SpC | 0.0 | 1.6 | 0.0 | 1.2 | 0.1 | 0.6 ± 0.8 | 0.1 | 1.4 | 0.1 | 8.9 | 0.2 | 2.1 ± 3.8 | 0.1 | 7.0 | 0.6 | 2.4 | 0.4 | 2.1 ± 2.9 |
| 1×10^7^ BMC + 3×10^7^ SpC + MAX.16H5 IgG_1_ | 0.9 | - | 0.5 | 0.5 | 1.1 | 0.8 ± 0.3 | 1.5 | - | 2.4 | 2.4 | 0.5 | 1.7 ± 0.9 | 0.7 | - | 0.4 | 1.8 | 0.5 | 0.8 ± 0.6 |
| 1×10^7^ BMC + 3×10^7^ SpC + 5×10^3^ 32D-FLT3^ITD^ | 0.1 | 3.9 | 0.1 | 1.0 | 0.2 | 1.1 ± 1.6 | 0.1 | 0.4 | 0.3 | 0.2 | 0.3 | 0.3 ± 0.1 | 0.3 | 3.4 | 0.3 | 1.3 | 0.6 | 1.2 ± 1.3 |
| 1×10^7^ BMC + 3×10^7^ SpC + 5×10^3^ 32D-FLT3^ITD^ + MAX.16H5 IgG_1_ | 4.2 | 0.3 | 10.6 | 0.2 | 19.6 | 7.0 ± 8.2 | 21.9 | 0.4 | 5.8 | 0.1 | 2.9 | 6.2 ± 9.1 | 4.7 | 0.7 | 8.8 | 1.6 | 3.6 | 3.9 ± 3.2 |

## Supplementary figures


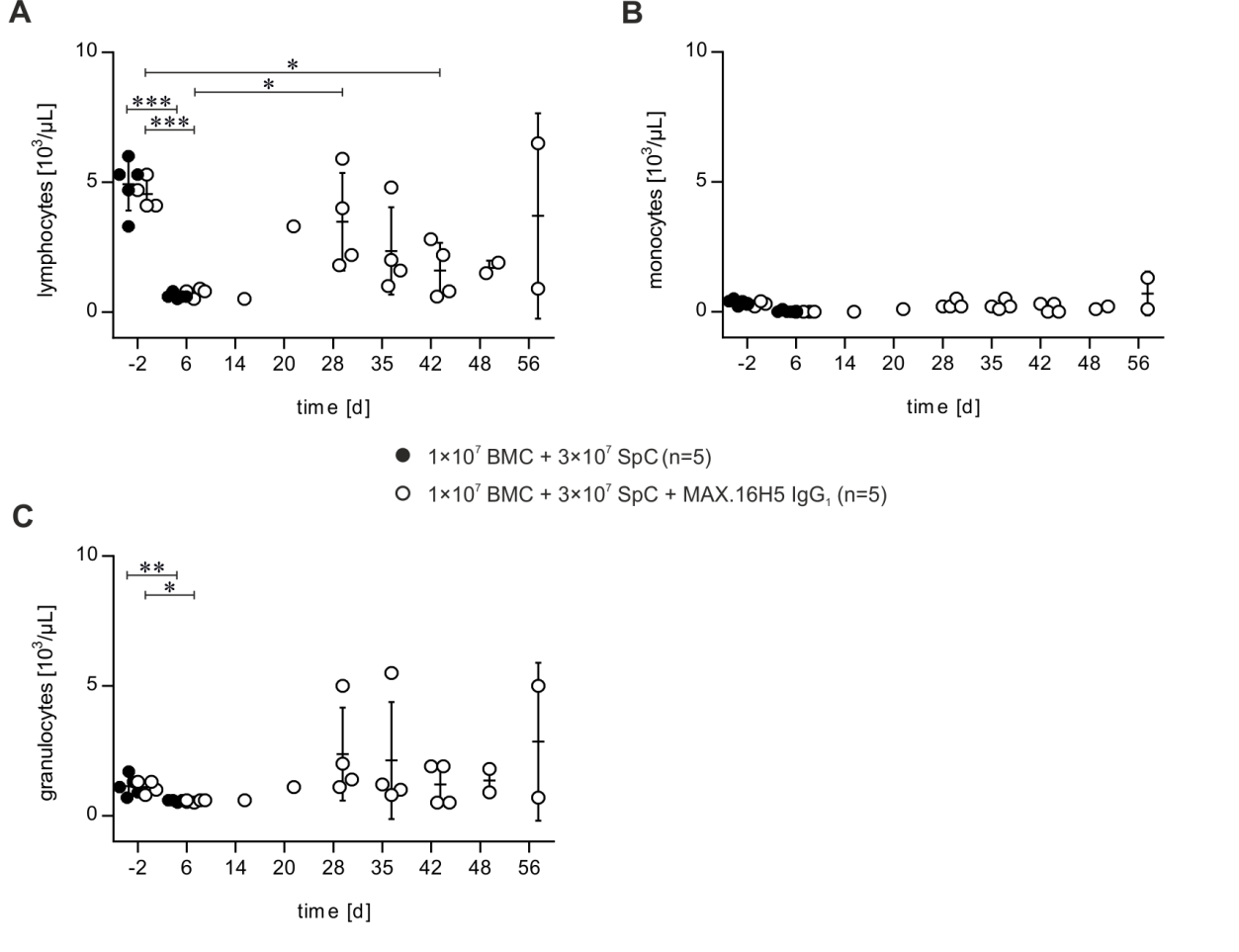


**Figure S1.** Full blood cell count in C3H/HeN mice after transplantation of 1×10^7^ bone marrow cells (BMC) and 3×10^7^ spleen cells (SpC) with MAX.16H5 IgG_1_ or without antibody short-term pre-incubation. All C3H/HeN mice received 1×10^7^ bone marrow and 3×10^7^ spleen cells as allogeneic transplant in these experiments. **(A)** Reconstitution of lymphocytes, **(B)** monocytes, and **(C)** granulocytes of C3H/HeN mice receiving either MAX.16H5 IgG_1_ pre-incubated grafts or grafts without prior antibody incubation. Each symbol indicates the value from one animal at a distinct time point. The error bars indicate mean values ± standard deviation. Contrasts only taking minimum sample sizes of four into account were pre‑defined and simultaneously tested for general linear hypothesis with Bonferroni adjustment. Monocyte numbers were not investigated, as the values were consistently lower in the global comparison with lymphocyte and granulocyte numbers. **(A)** Closed circles, day -2 vs day 6, P<0.001; open circles, day -2 vs day 6, P<0.001; day-2 vs day 42, P=0.012; day 6 vs day 28, P=0.025. **(C)** Only data sets from days -2 and 6 were compared. Closed circles, day -2 vs day 6, P=0.007; open circles, day -2 vs day 6, P=0.030.


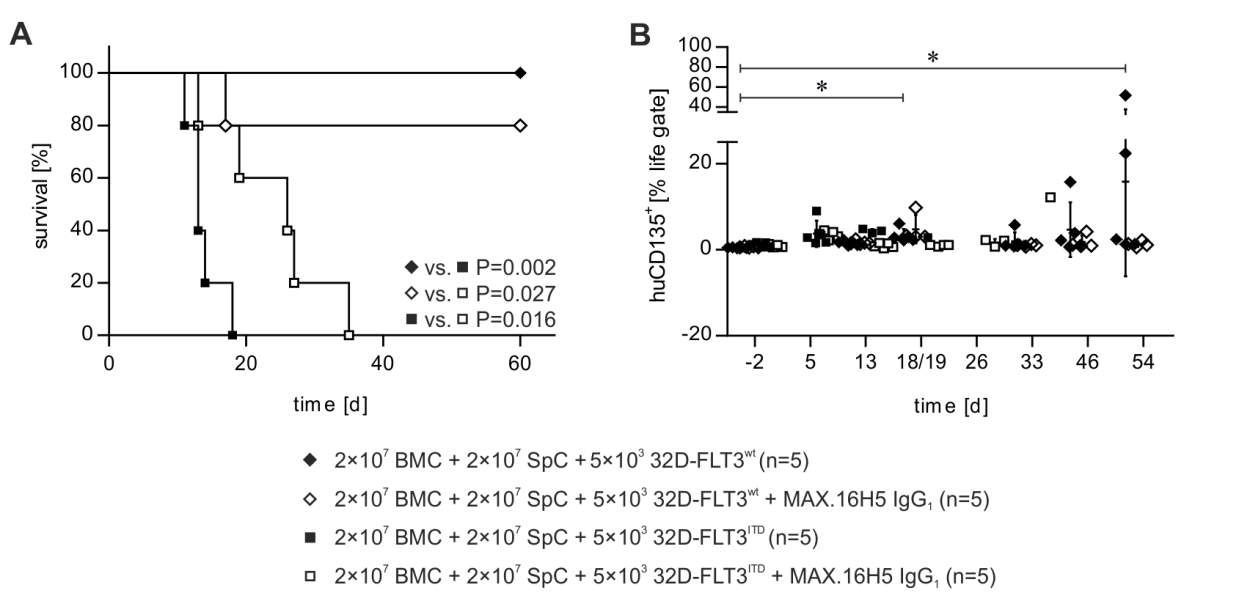


**Figure S2.** Survival analyses and detection of human CD135^+^ events by flow cytometry after transplantation of 2×10^7^ bone marrow cells (BMC) and 2×10^7^ spleen cells (SpC) either with or without prior MAX.16H5 IgG_1_ incubation and additional co-transplantation of either 5×10^3^ 32D-FLT3^wt^ or 5×10^3^ 32D-FLT3^ITD^ AML cells. All C3H/HeN mice received 2×10^7^ bone marrow and 2×10^7^ spleen cells as allogeneic transplant in these experiments. The grafts were pre-incubated either with or without MAX.16H5 IgG_1_ before administration into the recipients as well as in combination with transplantation of 5×10^3^ 32D-FLT3^wt^ or 32D-FLT3^ITD^ cells. **(A)** Survival of recipient mice after allogeneic transplantation. Animals receiving the 32D-FLT3^wt^ cells (each group n=5, diamonds) showed a significantly prolonged survival compared to animals receiving the 32D-FLT3^ITD^ cells (each group n=5, squares) (without MAX.16H5 IgG_1_: P=0.002, with MAX.16H5 IgG_1_: P=0.027, Log-Rank tests). Additionally, animals receiving the 32D-FLT3^ITD^ cells in combination with the MAX.16H5 IgG_1_ pre-incubated grafts (n=5, open squares) showed a significantly prolonged survival (until day 35) compared to the recipients of the untreated grafts (P=0.016, n=5, closed squares, Log-Rank test), which died within 18 days. **(B)** Flow cytometric data regarding the occurrence of human CD135^+^ events in whole blood samples of C3H/HeN recipient mice before and after allogeneic transplantation of bone marrow and spleen cell grafts either after a short-term pre-incubation with MAX.16H5 IgG_1_ or without antibody pre-incubation together with co-transplantation of 5×10^3^ 32D-FLT3^wt^ or 32D-FLT3^ITD^ AML cells. Mice receiving an antibody untreated graft in combination with 5×10^3^ 32D-FLT3^wt^ (n=5, closed diamonds) had increased human CD135^+^ counts indicating tumor engraftment. Data represent means ± standard deviations. Nemenyi tests with Bonferroni correction computed the comparative statistics. Only data sets consisting of at least four samples were considered. Closed diamonds, day -2 vs day 18, P=0.027; day -2 vs day 54, P=0.010.

**
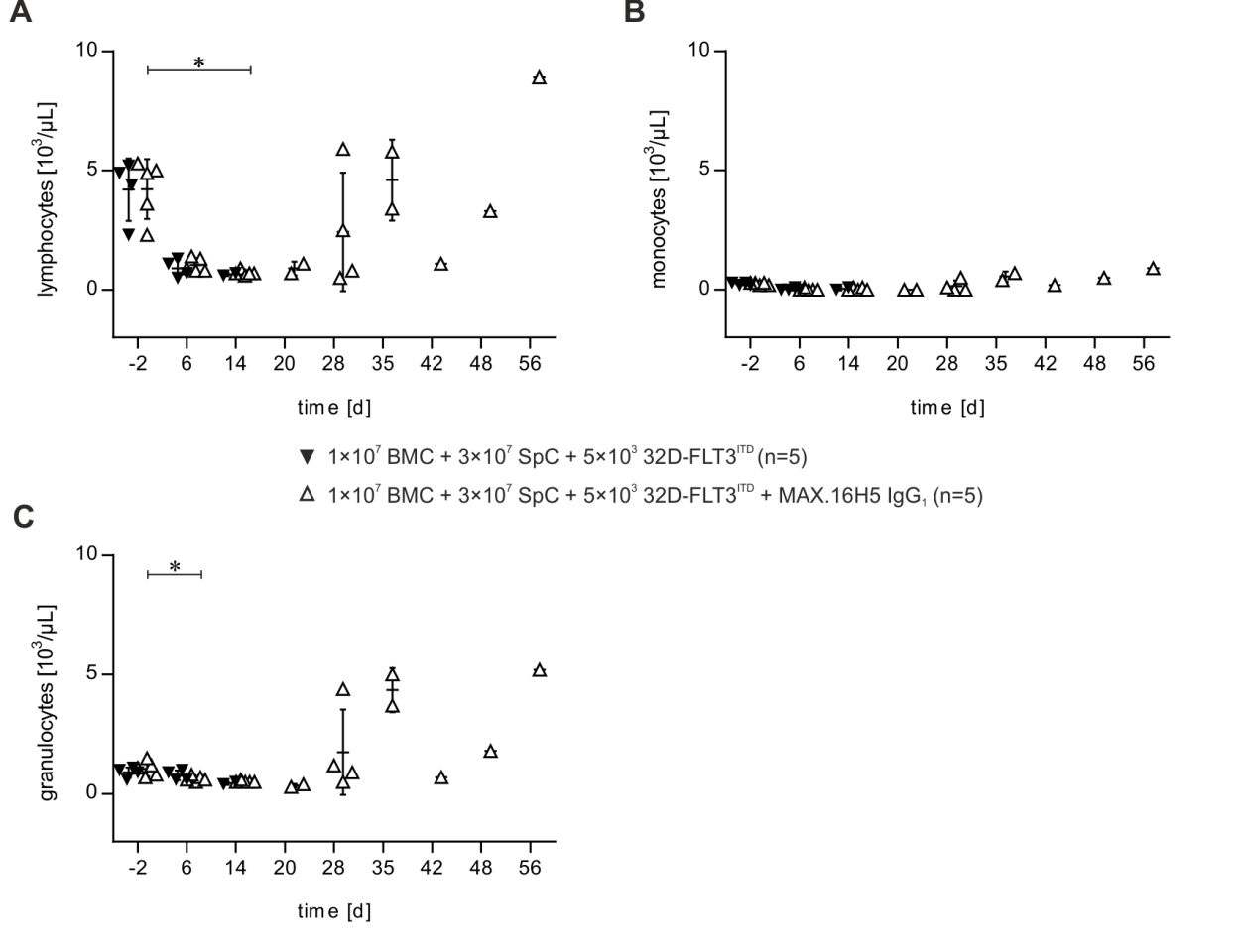
**

**Figure S3.** Full blood cell counts in C3H/HeN mice after transplantation of bone marrow cells (BMC) and spleen cells (SpC) with or without MAX.16H5 IgG_1_ short-term pre-incubation in combination with 5×10^3^ 32D-FLT3^ITD^ cells. All C3H/HeN mice received 1×10^7^ bone marrow and 3×10^7^ spleen cells as allogeneic transplants together with 5×10^3^ 32D-FLT3^ITD^ cells in these experiments. **(A)** Reconstitution of lymphocytes, **(B)** monocytes, and **(C)** granulocytes of C3H/HeN mice receiving either MAX.16H5 IgG_1_ pre-incubated grafts (n=5, open triangle) or grafts without prior antibody incubation (n=5, closed triangle). The symbols indicate individual values on distinct time points. The data points indicate mean values ± standard deviation. **(A)** Nemenyi tests with Bonferroni correction computed the comparative statistics. Only data sets consisting of at least four samples were considered. Open triangles, day -2 vs day 14, P=0.013. **(B)** Monocyte numbers were not investigated, as the values were consistently lower in the global comparison with lymphocyte and granulocyte numbers. **(C)** Only data sets from days -2 and 6 were compared in pre‑defined contrasts and simultaneously tested for general linear hypothesis with Bonferroni adjustment. Open triangles, day -2 vs day 6, P=0.046.


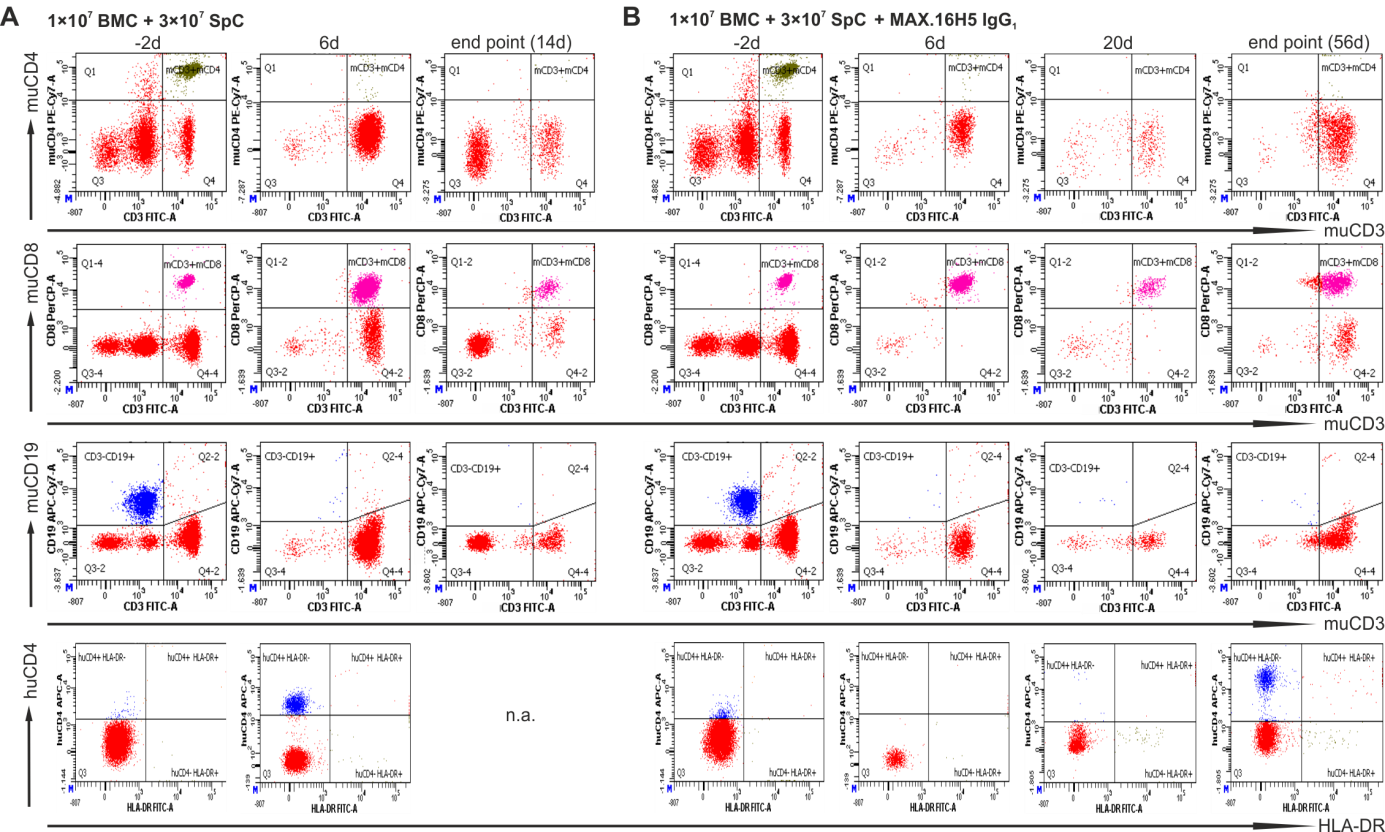


**Figure S4.** Graphs obtained from flow cytometric analyses of the immune cell reconstitution of murine CD3^+^, murine CD4^+^, murine CD8^+^, murine CD19^+^, and human CD4^+^ events after transplantation of bone marrow cells (BMC) and spleen cells (SpC) with or without short-term pre-incubation with MAX.16H5 IgG_1_. Representative scatter plots of flow cytometry measurements from one animal out of five are shown. All C3H/HeN mice received 1×10^7^ bone marrow and 3×10^7^ spleen cells as allogeneic transplant in these experiments. Whole blood samples were examined by flow cytometry at different time points (days -2, 6, 20, and endpoint) by gating for murine CD3/murine CD4, murine CD3/murine CD8, murine CD3/murine CD19 and HLA‑DR/huCD4 specific antibodies. **(A)** Blood sample from a mouse which received an antibody untreated graft and **(B)** from a mouse which received a MAX.16H5 IgG_1_ pre-incubated graft.

**
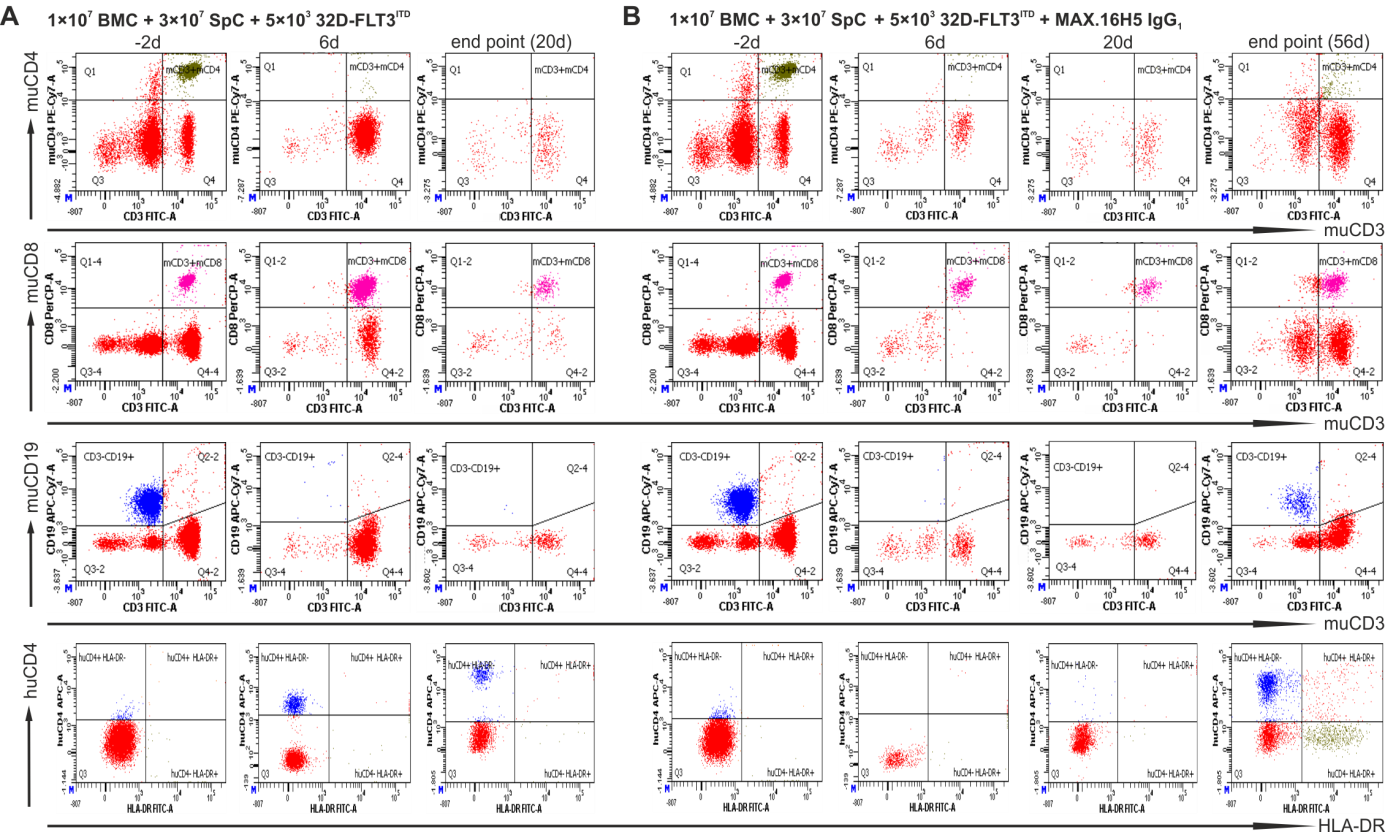
**

**Figure S5.** Graphs obtained from flow cytometric analyses of the immune cell reconstitution of murine CD3^+^, murine CD4^+^, murine CD8^+^, murine CD19^+^ and human CD4^+^ events after transplantation of bone marrow cells (BMC) and spleen cells (SpC) with or without short-term pre-incubation with MAX.16H5 IgG_1_ in combination with 5×10^3^ 32D-FLT3^ITD^ cells. Representative scatter plots of flow cytometry measurements from one animal out of five are shown. All C3H/HeN mice received 1×10^7^ bone marrow and 3×10^7^ spleen cells as allogeneic transplant together with 5×10^3^ 32D-FLT3^ITD^ cells in these experiments. The whole blood samples were examined by flow cytometry at different time points (days -2, 6, 20, and endpoint) by gating for murine CD3/murine CD4, murine CD3/murine CD8, murine CD3/murine CD19 and HLA‑DR/huCD4 specific antibodies. **(A)** Data from the blood from one mouse, which received an antibody untreated graft, and **(B)** from one mouse which received a MAX.16H5 IgG_1_ pre-incubated graft.

**
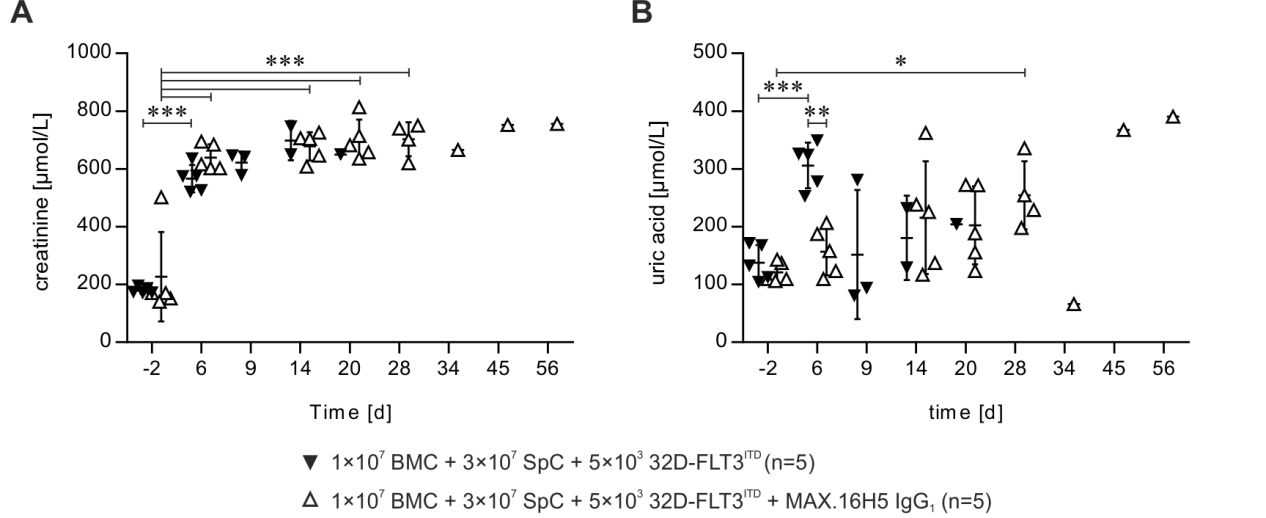
**

**Figure S6**

Analysis of laboratory parameters (creatinine and uric acid) from the plasma of transplanted mice. The plasma of mice receiving either 1×10^7^ bone marrow cells (BMC) and 3×10^7^ spleen cells (SpC) after a short-term pre-incubation with MAX.16H5 IgG_1_ or an untreated graft in combination with 5×10^3^ 32D-FLT3^ITD^ cells were examined regarding creatinine and uric acid concentrations. **(A)** The data show a significant increase in creatinine concentrations in both groups on day 6 in comparison to the concentrations on day -2 (without antibody: P<0.001 (n=5); with antibody: P<0.001 (n=5)). **(B)** The changes in the concentrations were recorded over a 56-day timespan. A significant increase in the uric acid concentrations was only observed on day 6 in mice receiving a graft without antibody pre-incubation (P<0.001, n=5). On day 6, the uric acid concentrations in the plasma from the group receiving MAX.16H5 IgG_1_ pre-incubated grafts significantly differed from those from the group receiving untreated grafts (P=0.003, n=5). Contrasts only taking minimum sample sizes of four into account were pre‑defined and simultaneously tested for general linear hypothesis with Bonferroni adjustment.

# Supplementary materials and methods

## Measurement of laboratory parameters

For the analyses of calcium, creatinine, phosphate and uric acid concentration the AU480^®^ Chemistry Analyzer (Beckman Coulter, Krefeld, Germany) was used as described earlier (1). At different time points of the experiment, plasma was collected by centrifugation at 300×g for 10 minutes and stored at ‑20°C. The plasma was diluted 1:20 with distilled water. The measurement was performed according to the manufacturer’s instructions. Concentrations below the detection limit of the system (negative results) were truncated and set to zero.

REFERENCES

1. Hilger N, Glaser J, Müller C, Halbich C, Müller A-M, Schwertassek U, et al. Attenuation of graft-versus-host-disease in NOD scid IL-2Rgamma(-/-) (NSG) mice by ex vivo modulation of human CD4(+) T cells. *Cytometry A* (2016) **89**(9):803–15.
